# Surveying cephalopod diversity of the Amazon reef system using samples from red snapper stomachs and description of a new genus and species of octopus

**DOI:** 10.1038/s41598-019-42464-8

**Published:** 2019-04-11

**Authors:** João Bráullio de Luna Sales, Manuel Haimovici, Jonathan Stuart Ready, Rosália Furtado Souza, Yrlene Ferreira, Jessica de Cassia Silva Pinon, Luis Fernando Carvalho Costa, Nils Edvin Asp, Iracilda Sampaio, Horacio Schneider

**Affiliations:** 1 Universidade Federal do Pará, Campus Universitário do Marajó-Breves, Faculdade de Ciências Naturais (FACIN), ZIP: 68800-000 Breves, PA Brazil; 2 Universidade Federal do Rio Grande, Laboratório de Recursos Demersais e Cefalópodes, ZIP: 96201-900 Rio Grande, RS Brazil; 3 Laboratório de Lepidopterologia e Ictiologia Integrada, Centro de Estudos Avançados da Biodiversidade, ICB-UFPA, ZIP: 66075-110 Belém, PA Brazil; 4 Universidade Federal Rural da Amazônia (UFRA), Belém, PA Brazil; 5 Laboratório de Filogenômica e Bioinformática, Instituto de Estudos Costeiros, Universidade Federal do Pará, Campus Universitário de Bragança, ZIP: 68600-000 Bragança, PA Brazil; 6 Universidade Federal do Pará, Instituto de Educação Matemática e Cientifica, Programa de Pós-Graduação em Educação em Ciências Matemáticas, ZIP: 66075-110 Belém, PA Brazil; 7 Laboratório de Genética e Biologia Molecular, Universidade Federal do Maranhão (UFMA), Centro de Ciências Biológicas e da Saúde, Departamento de Biologia, Campus Bacanga, São Luis, MA Brazil; 8 Laboratório de Geologia Costeira, UFPA-IECOS, Campus de Bragança, ZIP: 68600-000 Bragança, PA Brazil

## Abstract

The cephalopod fauna of the southwestern Atlantic is especially poorly-known because sampling is mostly limited to commercial net-fishing operations that are relatively inefficient at obtaining cephalopods associated with complex benthic substrates. Cephalopods have been identified in the diets of many large marine species but, as few hard structures survive digestion in most cases, the identification of ingested specimens to species level is often impossible. Samples can be identified by molecular techniques like barcoding and for cephalopods, mitochondrial 16S and COI genes have proven to be useful diagnostic markers for this purpose. The Amazon River estuary and continental shelf are known to encompass a range of different substrates with recent mapping highlighting the existence of an extensive reef system, a type of habitat known to support cephalopod diversity. The present study identified samples of the cephalopod fauna of this region obtained from the stomachs of red snappers, *Lutjanus purpureus*, a large, commercially-important fish harvested by fisheries using traps and hook-and-line gear that are capable of sampling habitats inaccessible to nets. A total of 98 samples were identified using molecular tools, revealing the presence of three squid species and eight MOTUs within the Octopodidae, representing five major clades. These include four known genera, *Macrotritopus*, *Octopus*, *Scaeurgus* and *Amphioctopus*, and one basal group distinct from all known octopodid genera described here as *Lepidoctopus joaquini* Haimovici and Sales, new genus and species. Molecular analysis of large predatory fish stomach contents was found to be an incredibly effective extended sampling method for biodiversity surveys where direct sampling is very difficult.

## Introduction

At least 800 species of cephalopods are known to exist worldwide^1,2^, and an increasing number of taxa are being described^3–7^, due largely to the application of new approaches, including genetic techniques, to identify cryptic species^8–10^. However, the difficulties of collecting cephalopod specimens are a major limitation for research. Most commercial fisheries are relatively ineffective at the capture of the majority cephalopod species, and in many cases, cephalopods are found in habitats that are difficult to fish^11,12^.

To circumvent these limitations, studies of the diets of the predators of cephalopods, including large fishes, marine mammals, seabirds, turtles, and even other cephalopods, can provide useful information on the occurrence of cephalopod species^13–16^. The identification of cephalopods found in stomach contents is hampered by the fact that their bodies are composed primarily of soft tissue, which is degraded rapidly by stomach acids. While the chitinous beaks are resistant to this acid, they typically permit identification only to genus or family level, making it impossible to differentiate sympatric congeneric species^17,18^. The identification of species can be achieved by using molecular techniques^19–21^, which have been used successfully when samples cannot be identified reliably based on their morphological characteristics.

In this context, DNA barcoding, which uses standardized protocols for the sequencing of a short fragment of the mitochondrial Cytochrome Oxidase I (COI) gene^22^, has proven to be very effective for the identification of species in many taxonomic groups including butterflies^23^, birds^24^, and plants^25^, as well as cephalopods^26^. In addition to the COI gene fragment, the large ribosomal subunit (16S rDNA) has also proven to be useful for the identification of cephalopod species at the intra-familial level^9,27^.

In northern Brazil, as in most of the tropical Atlantic, there are no fisheries specialized in harvesting cephalopods. Given this, samples are normally obtained as by-catch, usually from shrimp fisheries operating trawls over soft substrates, which can be an inefficient collection method for a variety of benthic cephalopod taxa. The present study focused on the poorly-known cephalopod fauna of the region of the Amazon Reef System (ARS), off the coast of northern Brazil^28^. This system was originally estimated to encompass an area of 9,500 km^2^, but it may be as large as 50.000 km^2^, and includes complex bottom habitats^29^, including biogenic reefs and basement outcrops, which are difficult to fish using nets. The basic consolidated substrates in the ARS are rhodolith beds, which might build extensive pavements. These pavements are often covered (and exposed) by sand wave migration, resulting in three-dimensional complexity. These features are intensively colonized by sponges, which results in additional complexity. Furthermore, fishes (e.g. Holocentridae Richardson, 1846) are excavating and reworking partially sand-recovered rhodolith beds, creating void spaces and building rhodolith mounds. Both voids and mounds are potential habitats for octopuses. On the other hand, the history of sea-level fall and rise over the Amazonian shelf has resulted in alternating deposition, formation and erosion of sedimentary deposits and rocks, where laterite outcrops seem to be particularly frequent in the southern sector of the ARS, offering abundant habitats for octopuses.

The study was based on the identification of the cephalopods found in the stomachs of red snapper, *Lutjanus purpureus* (Poey, 1875), a commercially important fish associated with these bottom habitats, which is harvested using traps and hook-and-line techniques. The red snapper is a large fish, which reaches 1 m in length and up to 10 kg in weight and is an active predator of benthic invertebrates. As it also tends to swallow its prey items whole, red snapper was selected as a likely candidate that could be used as an effective biological sampling tool for cephalopods in habitats that are difficult to survey^30–33^.

In this paper we describe the diversity of cephalopods found in the region, including a new genus and a new species of octopus that is both morphologically and genetically distinct from other genera and species in the Octopodidae.

## Results

### Genetic characterization

#### Squids

All squids sampled in this study (N = 14, 100%) were successfully amplified for the chosen fragment of the 16S rDNA gene but could not be amplified for the COI gene fragment. Analyses of squid data was therefore limited to 16S sequences. The GTR + I + G (−InL = 2738.3035) (AIC) and GTR + G (−InL = 2740.2735) (BIC) evolutionary models were selected by jModelTest2 for ML and BI analyses respectively. Comparisons with the GenBank sequences (selected using BLAST) associated the specimens to three species in the genera *Doryteuthis* Naef, 1912 and *Abralia* Gray, 1849 (Supplementary Data 1, Fig. 1). The majority (N = 12) of the individuals were genetically similar to *Doryteuthis plei* Blainville, 1823 (0.0–0.5% uncorrected p-distance), a species found on the Atlantic coast of Brazil^9,34^ (Supplementary Data 1). One specimen was genetically identical to samples identified as *Doryteuthis pealeii* Lesueur, 1821 (0.0% p-distance). A member of a probable species complex within *D. pealeii* is also found on the Atlantic coast of Brazil^9^. The last individual could only be assigned to the genus *Abralia* (grouping with other members in a clade with strong support: NJ/ML/BI 91%/88%/0.96) but was distinct from all the 16S sequences of *Abralia* species available in GenBank, with uncorrected p-distance ranging from 4.6% in relation to *Abralia adamanica* Goodrich, 1896 to 6.2% in the case of *Abralia trigonura* Berry, 1913 (Supplementary Data 1).

**Figure 1:**
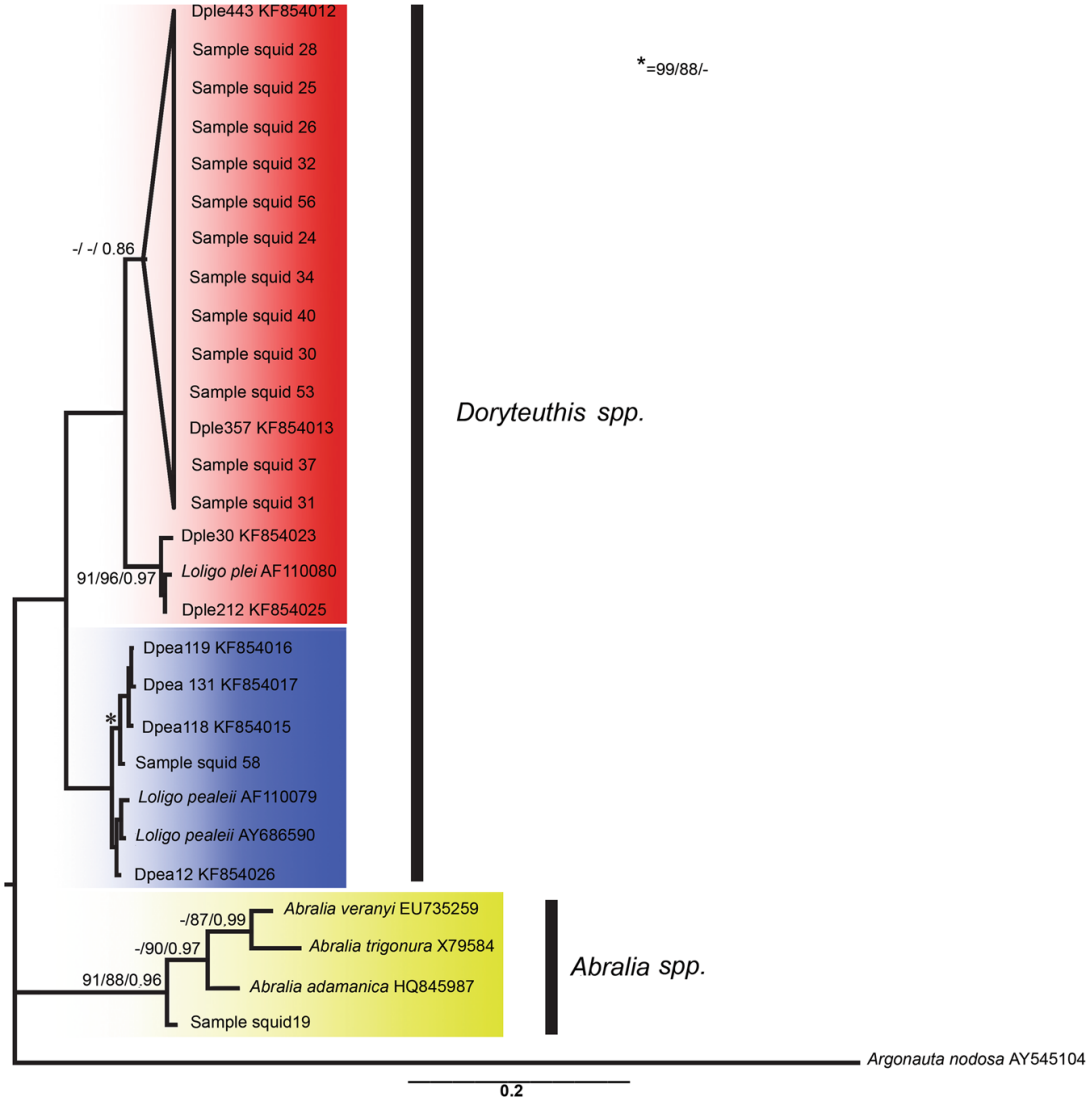
Phylogenetic tree based on Bayesian Inference of the mitochondrial 16S rDNA gene of the squid collected from the stomachs of the red snappers sampled in the present study. The values at the nodes show the bootstrap percentage for the Neighbor-Joining and Maximum Likelihood analyses, and *a posteriori* probabilities of Bayesian Inference analysis, respectively. Only values above 70%/0.7 are shown.

#### Octopods

**Figure 2:**
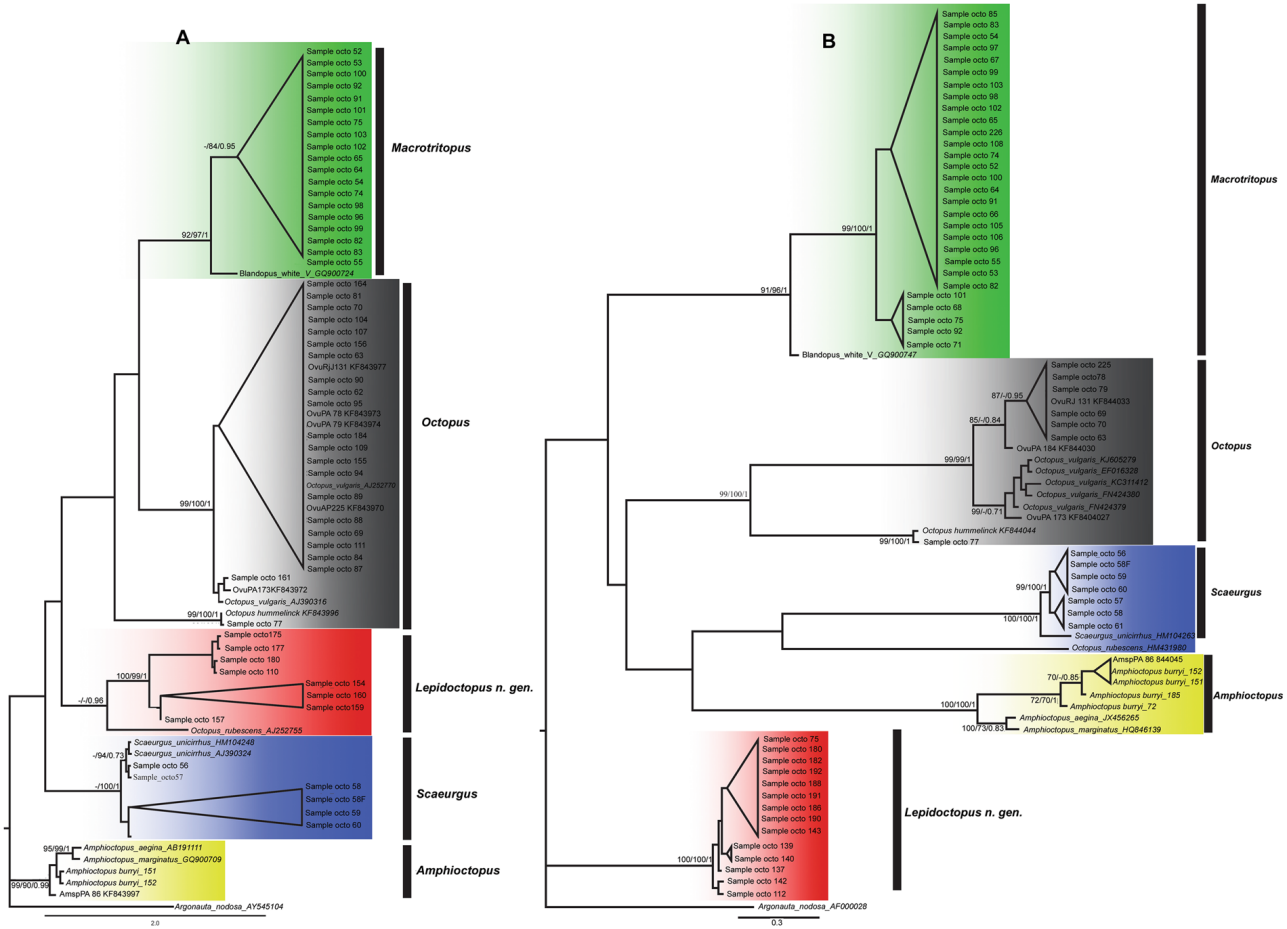
(**A**) Phylogenetic tree based on Bayesian Inference of the mitochondrial 16S r DNA gene of the octopuses collected from the stomachs of the red snappers sampled in the present study. The values at the nodes show bootstrap percentage for the Neighbor-Joining and Maximum Likelihood analyses, and *a posteriori* probabilities of the Bayesian Inference analysis, respectively. Only values above 70%/0.7 are shown. (**B**) Phylogenetic tree based on Bayesian Inference for the analysis of the mitochondrial COI gene of the octopuses, showing the same parameters analyzed for the 16S gene. Green clade: *Macrotritopus* sp. and most similar sequence from GenBank. Grey clade: *Octopus vulgaris* species complex and most similar sequences from GenBank. Red clade: *Lepidoctopus joaquini* gen. et sp. nov. and most similar sequence from GenBank. Blue clade: *Scaeurgus* sp. and most similar sequences from GenBank. Yellow clade: *Amphioctopus* sp. and most similar sequences from GenBank.

Of the 130 sampled octopods, 84 were successfully sequenced for one or another target sequence, including 61 (~73%) for 16S rDNA and 61 (~73%) for COI. Unfortunately, successful amplification of both target sequences was only possible for 38 samples (Supplementary Data 2). For the 16S sequences, the evolutionary models selected were GTR + I + G (−InL = 2738.3035) (AIC) for ML analysis, and GTR + G (−InL = 2740.2735) (BIC) for BI analysis. In the case of the COI gene, the TIM2 + I + G (−InL = 3313.5079) model was selected (AIC and BIC) for both the ML and the BI analyses. Five principal monophyletic groups were recovered in these analyses, including the four genera, *Macrotritopus* Grimpe, 1922*, Octopus* Cuvier, 1797 (but note that *Octopus* itself is not monophyletic), *Scaeurgus* Troschel, 1857 and *Amphioctopus* Fischer, 1882, and a fifth group distinct from all octopodid genera that have sequences in GenBank (Fig. 2A).

The samples identified morphologically as *Macrotritopus* sp., grouped with sequences that have been given the name “Blandopus white V” in the phylogenies for both the 16S dataset (4.4–5.2% uncorrected p-distance) and the COI dataset, with 4.9–5.5% uncorrected p-distance (Supplementary Data 2A,B, Fig. 2). In the case of the samples identified as *Octopus*, the two lineages found in the 16S dataset were most closely associated with sequences published in GenBank as *Octopus vulgaris* Cuvier, 1797 from Brazil (KF843970/KF843972/KF843973/KF843974/KF843977) and from Venezuela (AJ252770/AJ390316). Although there was some overlap, within lineage uncorrected p-distance (0.0–0.9%, mean = 0.3%) was lower than between lineage uncorrected p-distance (0.5–1.6%, mean = 1.1%). For COI there was no overlap. Within lineage uncorrected p-distances were all 0.0%. A 0.5% distance exists between the first lineage containing only Brazilian samples and a second lineage containing the sample OvuPA184 (sequence KF844030). Both lineages were yet more divergent (2.1% uncorrected p-distance for the first, and 1.6% uncorrected p-distance for the second) from a third lineage including the Brazilian sample OvuPA173 (sequence KF844027) and other GenBank sequences (Fig. 2 Supplementary Data 3C,D). This indicates the presence of distinct haplogroups in the region that unfortunately cannot be associated to the described Types I or II^35^ due to the state of the voucher material. Furthermore, another sequence obtained in the present study was identical to the sequence of a sample previously identified as *Octopus hummelincki* Adam, 1936 from the northeastern Brazilian state of Ceará (0.0% uncorrected p-distance for both 16S and COI sequences).

The samples identified as *Scaeurgus unicirrhus* Delle Chiaje, 1839–1841 are represented by two lineages with low divergence (uncorrected p-distances of 0.0–1.2% for 16S and 0.0–1.0% for COI, Supplementary Data 2E,F). One of the lineages is more closely related to GenBank sequences for *Scaeurgus unicirrhus* based on 16S sequences, but both lineages were more closely related to each other and approximately equidistant from GenBank sequences of *S. unicirrhus* based on COI data, (Fig. 2B), indicating the likely existence of distinct stocks within the species.

For the genus *Amphioctopus* new sequences formed a strongly-supported monophyletic group along with available GenBank sequences (Fig. 2B, node with NJ/ML/BI support 99%/99%/0.99), and uncorrected p-distance values of between 0.5–2.74% for 16S and 0.2–4.2% for COI (Supplementary Data 2G,H) within the clade. These specimens were difficult to identify morphologically due to their degraded condition, but they could be identified based on their COI sequences. Two of the samples were genetically identical to AmspPA86 (GenBank access code KF844045)^36^ and unpublished sequences of specimens collected offshore from the state of Rio de Janeiro, in southeastern Brazil, which were identified morphologically as *Amphioctopus burryi* Voss, 1950 (M. Haimovici; unpublished data).

The fifth group of specimens could not be associated clearly with any known genus (herein described as *Lepidoctopus* gen. nov.). Some individuals were linked weakly with GenBank sequences named as *Octopus rubescens* Berry, 1953 based on the 16S data (BI = 0.96), but the uncorrected p-distance between these sequences was 12.0–12.2% (Fig. 2A, Supplementary Data 2G).

**Figure 3:**
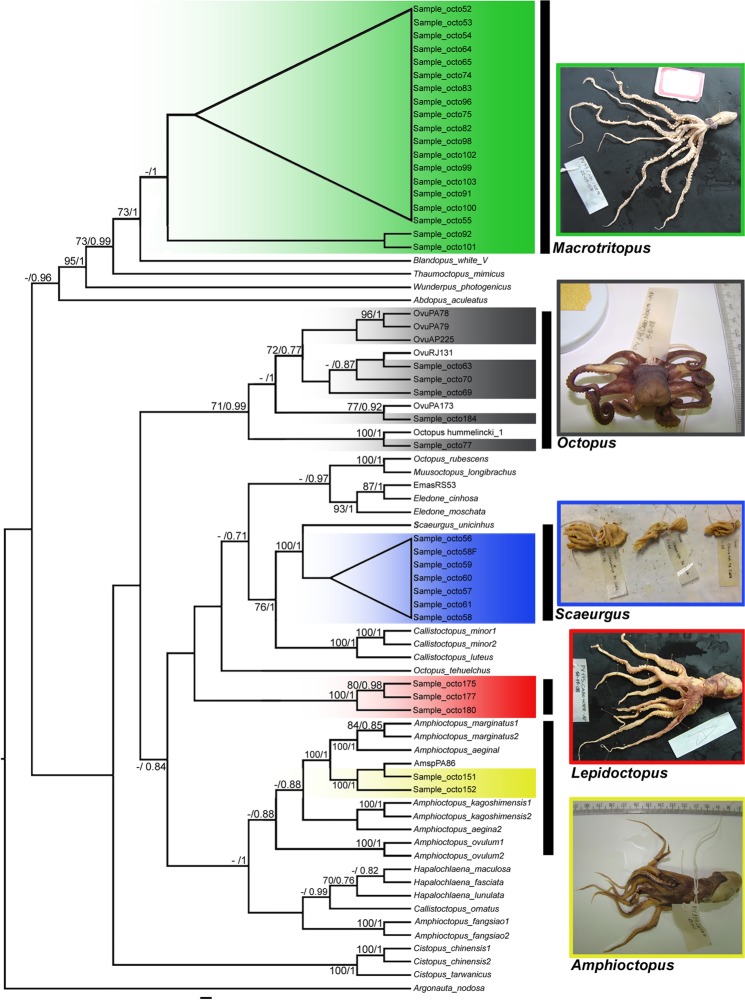
Phylogenetic tree based on Bayesian Inference for the concatenated 16S + COI data set of the octopuses analyzed in the present study. The values at the nodes refer to the bootstrap percentage for Maximum Likelihood and *a posteriori* probabilities of the Bayesian Inference analysis, respectively. Only values above 70%/0.7 are shown. Green clade: *Macrotritopus* sp., represented by sample octo 97 in photo. Grey clade: *Octopus* spp., represented by sample octo 148 in photo. Blue clade: *Scaeurgus* sp., represented by samples octo 58, 59 and 60 in photo. Red clade: *Lepidoctopus joaquini* gen. et sp. nov., represented by sample octo 175 in photo. Yellow clade: *Amphioctopus* sp., represented by sample octo 114 in photo.

The complete phylogeny, based on the samples with data for both markers (concatenated dataset), provides a more reliable overview of the arrangement of the principal genera within the family Octopodidae (Fig. 3). This analysis revealed that *Macrotritopus* is most closely related to the other mimetic forms (*Thaumoctopus* Norman & Hochberg 2005, “Blandopus”, and *Wunderpus* Hochberg, Norman & Finn, 2006), with *Abdopus aculeatus* d’Orbigny, 1834 as a probable basal member of the clade (Fig. 3). Once again, the group of genetically distinct individuals that could not be assigned to any genus in the previous analyses (herein described as *Lepidoctopus* gen. nov.) formed a highly divergent lineage, with no well-supported relationship with any genus for which sequences are available. The phylogeny (Fig. 3) indicates a polytomy including this divergent lineage and a miscellaneous group including the clades ((*Octopus rubescens* + *Muusoctopus longibrachus longibrachus* Ibãnéz, Sepúlveda & Chong, 2006) + *Eledone* Leach, 1817), (*Scaeurgus* + *Callistoctopus* Taki, 1964), *Octopus tehuelchus* d’Orbigny, 1834 and (*Amphioctopus* + *Hapalochlaena* Robson, 1929).

### Morphological characterization

#### Squids

Squid could only be identified to family level because of the loss of most morphological diagnostic characteristics of the samples.

#### Octopods

Based on external morphological features alone, only three groups could be identified in the 130 octopuses examined. Some specimens, including those identified based on sequence identity as *Scaeurgus unicirrhus* and *Amphioctopus burryi* were too degraded to confirm morphological identification.

#### Macrotritopus sp

Small octopus, the largest sampled specimen was 237 mm total length and 31 mm mantle length. Mature females bore a large number of 1–2 mm long oocytes. The mantle is oval, mantle width is 32–41% of the mantle length. The mantle is grayish dorsally and cream ventrally, the eyes are large and protuberant, 11–17% the mantle length, the funnel is moderately long with more than half its length free. The gills have 9–10 lamellae in each of the two hemibranchs. The web is short, and the arms long and thin with two series of relatively small suckers. The ventral arms are the longest and reach five times the length of the mantle. A single male had both third arms and the right hectocotylized arm was 76% as long as the left arm. Short hectocotylus (2% of the arm length) with a small calamus, and a ligula with a longitudinal groove. These characteristics are broadly consistent with the description of *Macrotritopus defilippi* (Verany, 1851)^37^ and of *Macrotritopus beatrixi* Guerrero-Kommritz & Rodriguez-Bermudez^38^. The known distribution of *Macrotritopus defilippi* is in the Mediterranean and northeastern Atlantic Ocean, while some authors apply the same name to specimens with similar characteristics from the Caribbean and southwestern Atlantic Ocean^39,40^ and *M. beatrixi* is only described for the Caribbean Sea along Colombia^38^. To elucidate if our specimens pertain to either species or to an undescribed third one requires more reliable morphological data for a formal description.

#### Octopus vulgaris species complex

Most specimens of this type were in poor condition. One very well-preserved individual, which unfortunately we were not able to sequence, coincided with the description of the *Octopus vulgaris* species complex.

### Specimens not associated with any known genus

Among the better-preserved specimens included a group of small octopuses with long arms and a moderately thick and short web. The mantle, head and base of the arms is covered with large rounded papillae, resembling dermal cushions, some of which have long conical cirri with multiple tips. In males, the third right hectocotylized arm has a very short calamus and a long, slender ligula with a deep longitudinal groove. This combination of characters was not recorded in any genus of the Family Octopodida^35^. We consider these specimens to be an undescribed species of a newly discovered genus, as follows:

Family Octopodidae d’Orbigny, 1840

Subfamily Octopodinae d’Orbigny, 1840

*Lepidoctopus joaquini* Haimovici and Sales, gen. et sp. nov.

Diagnosis of the genera and species**:** small sized benthic octopod, largest examined specimen 40 mm mantle length (ML); mantle, head and base of arms covered by large rounded papillae-like dermal cushions more densely packed and larger on dorsal mantle and smaller on head and web; some papillae on dorsal mantle bear cirri branched in multiple tips; no lateral ridge observed; eyes moderate in size, slightly protruding and with single supraocular papillae with large branched cirrus, funnel half of ML; arms long, first and second typically around 4.5 times ML, third and fourth under 4.0 times ML; web typically half of ML; normal arms with up to 170 suckers, first 4 to 6 proximal suckers in single series, followed biserial to tips of arms; enlarged suckers in fourth to sixth biserial rows of males; third left arm of males hectocotylized, ~77% of the opposite third arm with 68 to 72 suckers, Short hectocotylus, ~22% of hectocotylized arm, with short conical calamus, 2% of ligula length, slender ligula with deep longitudinal groove ending in blunt tip.

### Material examined

Eleven specimens, eight males and three females among the best-preserved specimens collected in stomach contents of the snapper *Lutjanus purpureus* commercially fished with a fish trap or lines off northern Brazil. All deposited in the mollusk collection of the Oceanography Museum of the University of Rio Grande (MORG) in Rio Grande, Rio Grande do Sul, Brazil.

#### Holotype

(Sample PV192) Mature male 28 mm ML, coastal waters off Cabo Norte, Amapá State, northern Brazil, 03°34′27″N, 50°03′49″W, 50–60 m. in 14 Dec. 2008. MORG coll. num. 51455. Molecular sequence: GenBank Accession No. MG010599 (COI)

#### Paratypes

Sample PV135 One mature male 36 mm ML, coastal waters off Pará State, northern Brazil, 00°05′53″N 47°14′08″W, 20–30 m in 17 Dec. 2007. MORG coll. num. 51448.

Sample PV137 One mature male 40 mm ML, coastal waters off Pará State, northern Brazil, 00°05′53″N 47°14′08″W, 20–30 m in 17 Dec. 2007. MORG coll. num. 51449. Molecular sequence: GenBank Accession No. MG010602 (COI).

Sample PV139 One mature male 33 mm ML, coastal waters off Pará State, northern Brazil, 00°05′53″N 47°14′08″W, 20–30 m in 17 Dec. 2007. MORG coll. num. 51450. Molecular sequence: GenBank Accession No. MG010603 (COI).

Sample PV142 One mature male 33 mm ML, coastal waters off Pará State, northern Brazil, 00°05′53″N 47°14′08″W, 20–30 m in 17 Dec. 2007. MORG coll. num. 51451. Molecular sequence: GenBank Accession No. MG010600 (COI).

Sample PV162 One mature male 29 mm ML, coastal waters off Pará State, northern Brazil, 00°05′53″N 47°14′08″W, 20–30 m in 12 Nov. 2008. MORG coll. num. 51452.

Sample PV182 One mature male 29 mm ML, coastal waters off Pará State, northern Brazil, 00°05′53″N 47°14′08″W, 20–30 m in 12 Nov. 2008. MORG coll. num. 51453. Molecular sequence: GenBank Accession No. MG010594 (COI).

Sample PV191 One mature male 31 mm ML, coastal waters off Pará State, northern Brazil, 00°05′53″N 47°14′08″W, 20–30 m in 14 Dec. 2008. MORG coll. num. 51454. Molecular sequence: GenBank Accession No. MG010598. (COI).

Sample PV175 One mature female 30 mm ML, coastal waters off Pará State, northern Brazil, 00°05′53″N 47°14′08″W, 20–30 m in 12 Nov. 2008. MORG coll. num. 51456. Molecular sequence: GenBank Accession No. MG010542 (16S) and MG010592 (COI).

Sample PV177 One mature female 33 mm ML, coastal waters off Pará State, northern Brazil, 00°05′53″N 47°14′08″W, 20–30 m in 12 Nov. 2008. MORG coll. num. 51457. Molecular sequence: GenBank Accession No. MG010543 (16S) and MG010602 (COI).

Sample PV188 One mature female 34 mm ML, coastal waters off Pará State, northern Brazil, 00°05′53″N 47°14′08″W, 20–30 m in 12 Nov. 2008. MORG coll. num. 51458.

### Description

The description is based on eight males and three females in initial stages of digestion. Most had the skin damaged and either the arm tips or distal suckers were missing from some arms including all non-hectocotylized arms. However, a sufficient number of arms and web depth could be measured to calculate approximately the arm and web depth formulas. The largest male is a mature specimen, 40 mm ML, 274 mm TL, 36.2 g TW, with fully developed spermatophores in the spermatophoric sac. The largest female in fair condition presented a large ovary occupying the posterior half of the mantle cavity and was full of developing eggs. This female measured 34 mm ML, 218 TL and weighed 19.9 g TW. Morphometric measurements are presented in Table 1 and morphometric indices in Table 2. Values in the text are reported as mean value and range.

**Table 1:** *Lepidoctopus joaquini* gen. et sp. nov.: Counts and measurements (millimetres).

Collection site	Bragança	Bragança	Bragança	Bragança	Cabo Norte	Cabo Norte	Cabo Norte	Cabo Norte	Cabo Norte	Cabo Norte	Cabo Norte
Gene Bank number	—	MG010602	MG010603	MG010600	—	MG010594	MG010598	MG010599	MG010542	MG010543	—
Catalog number (MORG)	51448	51449	51450	51451	51452	51453	51454	51455	51456	51457	51458
Status	Paratype	Paratype	Paratype	Paratype	Paratype	Paratype	Paratype	Holotype	Paratype	Paratype	Paratype
Collection date	17/12/2007	18/12/2007	19/12/2007	20/12/2007	21/12/2007	22/12/2007	23/12/2007	24/12/2007	25/12/2007	26/12/2007	27/12/2007
Sex	Male	Male	Male	Male	Male	Male	Male	Male	Female	Female	Female
Total fixed weight (g)	27.4	36.2	18.8	19.2	14.3	20	22.4	17.5	15.6	19.8	19.9
Total length	232	274	186	180			157	174	174		218
Dorsal mantle length	36	40	33	29	29	29	31	28	30	33	34
Mantle width	21	25	21	18	18	17	21	17	21	17	20
Head width	16	15	13	14	14	16	15		15	15	14
Mean eye diameter	4,0	5,0	4.5	5,0	4,0	5,0	4,0	5,0	3,0	4,0	5,0
Funnel length	17	17	17	15	14	14	15	15	15	15	18
Free funnel length	10	10	7	9	8	9	7	9	8	9	12
Paleal opening	17	23	17	13	20	15	14	14	16	15	15
Hectocotylus length	21	21		19			16	17.5			
Ligula length	20	20		18			15.5	16.5			
Calamus length	1	1		1			0.5	1			
Gill lamellae count (I/O R–I/O L)	7/7–7/7	7/7–7/7	7/7–7/7	7/7–7/7	7/7–7/7	7/7–7/7	7/7–	-/7–/7	7/7–7/7	7/7–7/7	-/7–7/-
Gill Length (mean)	15.5	13.5	13.5	11.0	13.0	12.0	10.0	9.0	11.5	12.5	13.0
Arm lengths 1 (R-L)	d	207 R	147 R					110 L		140–140	
Arm lengths 2 (R-L)	d	186–227			129 R		117 R	134–131	121 L	120–140	162 L
Arm lengths 3 (R-L)	150–184	138 Hc -196		95 R	112 R		92 Hc - 115	88 Hc	104 R	130–125	
Arm lengths 4 (R-L)	d	d			122 R		94 L		111 L	110–115	111 L
Arm width (mean)	5.2	5.1	5.0	5.0	4.0	4.7	4.6	4.3	4.3	4.3	4.0
Web Depth A (dorsal)	27	18				21	23	18	18		24
Web Depth B (R-L)	28 R	20–27	27 R			20–25	22 R	22–20.5	17 R		22 R
Web Depth C (R-L)	29–28	25–20				22–32	27–22	20 R			24 R
Web Depth D (R-L)	27 L	26 L				21–24	16 R	19–20			18 L
Web Depth E (ventral)	30		25						16		30
Larger sucker arm 1 (R-L)	4.9–3.8	3.1–3.8	5 R	4.3 R			3.8–3.7	3.2–3.4	2.9–2.0	2.6–2.6	1.7–1.8
Larger sucker arm 2(R-L)	4.3–3.8	4.2–3.1	4.6 R				4.0–3.7	3.6–2.7	2.7–2.4	2.4–2.5	1.7–2.0
Larger sucker arm 3 (R-L)	3.2–3.1	4.2–3.6	4.5 R	4.0–4.5			3.9–3.9	3.2–3.2	2.4–2.2	3.0–2.7	2.0–2.4
Larger sucker arm 4 (R-L)	2.3–2.5	3.3 L	4.3–4.0	3.8			3.8–4.1	2.4–2.5	2.2–2.1	2.5–2.4	1.7–2.3

R = right, L = left, I = inner, O = outer, Hc = hectocotylized arm, 1 to 4 arm numbers, A to E web depths.

**Table 2:** Measurements of *Lepidoctopus joaquini* gen. et sp. nov.: mantle width, head width, gill length, funnel length, eye diameter, arm lengths, web depth and larger sucker indices as percentage of the mantle length; hectocotylus length and ligula length as percentage of the hectocotylized arm, free funnel length as percentage of funnel length and calamus length as percentage of ligula length.

Catalog number (MORG)	51448	51449	51450	51451	51452	51453	51454	51455	51456	51457	51458
Status	Paratype	Paratype	Paratype	Paratype	Paratype	Paratype	Paratype	Holotype	Paratype	Paratype	Paratype
Sex	Male	Male	Male	Male	Male	Male	Male	Male	Female	Female	Female
Mantle width	58%	63%	64%	62%	62%	59%	68%	61%	70%	52%	59%
Head width	44%	38%	39%	48%	48%	55%	48%		50%	45%	41%
Gill length	43%	34%	41%	38%	45%	41%	32%	32%	38%	38%	38%
Funnel length	47%	43%	52%	52%	48%	48%	48%	54%	50%	45%	53%
Free funnel length	59%	26%	21%	31%	27%	29%	23%	32%	26%	26%	35%
Mean Eye diameter	76%	60%	62%	78%	78%	94%	71%	0%	71%	88%	70%
Arm 1 length		518%	445%					393%		424%	
Arm 2 length		568%			445%		377%	479%	403%	424%	476%
Arm 3 length	511%	490%		328%			371%	314%	347%	394%	
Arm 4 length		545%			421%		303%		370%	348%	326%
Arm width	14%	13%	15%	17%	14%	16%	15%	15%	14%	13%	12%
Web depth (A = dorsal)	75%	47%				72%	74%	64%	58%		69%
Web depth (B)	78%	53%	79%			69%	71%	79%	55%		
Web depth (C)	81%	66%				110%	87%	71%			
Web depth (D)	89%	68%				83%	52%	68%	51%		51%
Web depth (E = ventral)	83%		74%						52%		86%
Largest sucker males	10%	9%	14%	14%			12%	11%			
Largest sucker females									8%	8%	6%
Hectocotylus length	82%	70%					80%				
Ligula length	13%	14%		19%			17%	19%			
Calamus length	5%	5%		6%			3%	6%			

#### Morphology

**Figure 4:**
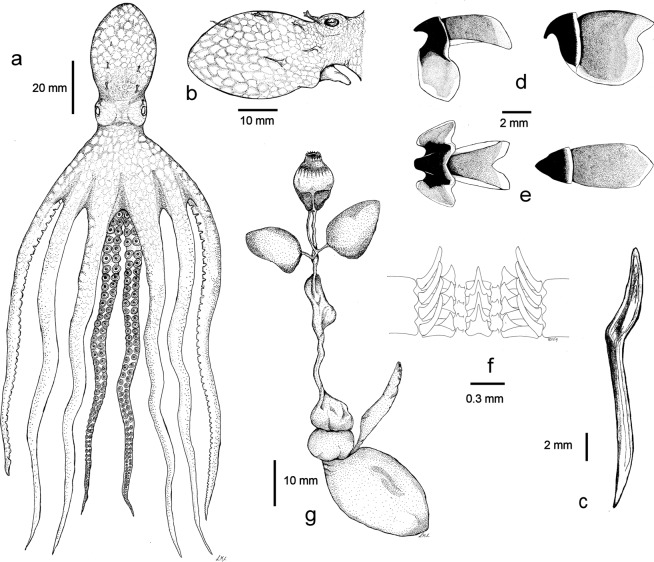
*Lepidoctopus joaquini* gen. et sp. nov.: (**a**) 40 mm mantle length male with reconstructed arms and sucker, (**b**) lateral view of the mantel and head with papillae and cirrus pattern. (**c**) Stylet. (**d**) Lateral view of lower (left) and upper (right) beaks. (**e**) Ventral view of lower beak (left) and dorsal view of upper beak (right), (**f**) radula. Each drawing has a specific scale bar. All imagens produced by contracted biological artist Leticia Cavole.

Mantle oval and elongate, width 61% (50–68%) of ML; head is narrower than mantle, 45% (38–55%) of ML (Fig. 4a); Mantle, head, and arms in preserved specimens reddish brown dorsally, lighter cream ventrally (Supplementary Data 3 and 4); Skin of mantle, head and base of arms sculptured with large rounded, densely packed papillae-like dermal cushions with chromatophores on borders (Supplementary Data 5); Papillae on dorsal mantle larger, some modified with long conical cirri branched in multiple tips; Papillae on ventral mantle smaller with fewer chromatophores (Fig. 4b); No lateral ridge observed.

Eyes large, 14% (10–18%) of ML, slightly protruding; Supraocular papillae present with large branched cirrus (Fig. 4b).

**Figure 5:**
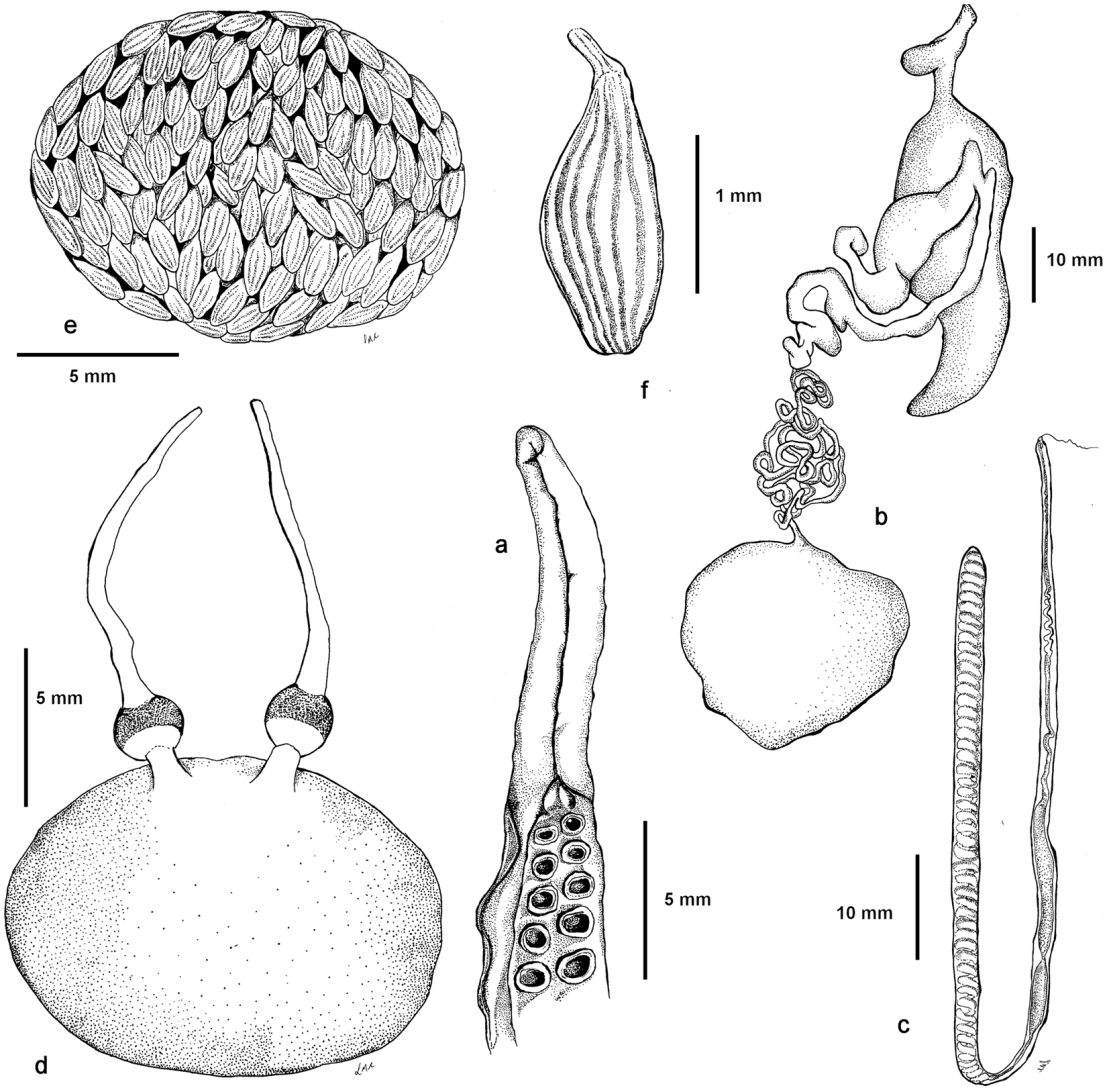
*Lepidoctopus joaquini* gen. et sp. nov.: (**a**) Copulatory organ. (**b**) Male reproductive tract. (**c**) Spermatophore. (**d**) Female reproductive tract. (**e**) Schematic view of maturing ovary. (**f**) Maturing oocyte. Each drawing has a specific scale bar. All imagens produced by contracted biological artist Leticia Cavole.

Stylets present in mantle muscle at base of gills; Stylets relatively long, 35% of ML, with angle at 1/3 of its length, shorter posterior limb thicker and with distinct deep groove in which growth lines observed (Fig. 4c); Funnel relatively long, 48% (44–53%) of ML, free for 56% (41–60%) of its length, reaching anterior margin of eyes; Funnel organ not observed; Arms long and rather stout; First, second and third typically around 4.5 times ML fourth around 3.5 times ML (arm formula: 1 = 2 = 3 > 4); Arm of moderate width, 14.3% (11.4–17.2%) of ML; No difference in width observed between arms within individuals or between sexes; Webs moderately short, 76% of ML; Web formula non-consistent between specimens; First 4 to 6 proximal suckers in single row followed by biserial suckers to tips of arms; Suckers moderately sized, larger suckers did not differ between arms in females, 7.2% (5.7–7.9%) of ML; In males, 4 to 6 enlarged suckers present on all arms 11.6% (9.0–14.3%) of ML; Suckers on distal half of arms much smaller than those in proximal half (Fig. 4a); Third left arm of males hectocotylized and shorter (Supplementary Data 6), 77% (70–81%) of third right arm; Hectocotylized arm bears 68 to 82 suckers; Counts of number of suckers of all other arms not accurate due to degradation; Three largest counts ranged from 164 to 170; Ligula long, 16% (13–18%) of hectocotylized arm, and slender with deep longitudinal groove, ending in blunt tip; Calamus conical, very small, 5.5% of ligula length (Fig. 5a); Gills relatively long, 32% to 43% of ML, 7 lamellae on both inner and outer demibranchs in all examined specimens (gill lamellae number formula: L = 7/7; R = 7/7); Upper beak robust, with hooked rostrum and moderately long hood, 34% of total beak length, lower beak with rounded rostrum and hood measuring 62% of crest length (Fig. 4d,e); Radula with seven teeth and two marginal plates in each transverse row (Fig. 4f); Rhachidian tooth with one to two lateral cusps, typically two, on each side of large medial cone; lateral cusps in symmetrical seriation, migrating from lateral to medial position over approximately five to six transverse rows.

#### Digestive tract

Anterior salivary glands typically discoid, relatively small, 1/3 length of buccal mass; Posterior salivary glands large, each approximately as big as buccal mass; Elongated crop with distinct diverticulum; Stomach and caecum of same size; Digestive gland ovoid to lobed; Ink sac present, embedded in digestive gland (Fig. 4g); Distal half of intestine of specimens not recovered, presence of anal flaps undetermined.

#### Male reproductive system

Testicles in mature specimens oval, vas deferens very long thin and coiled; First spermatophore gland long and thin and second spermatophore gland short; Spermatophore sac of mature males containing (30 to 50) fully formed spermatophores; Terminal organ in mature males small, curved and with moderately large diverticulum (Fig. 5b); Spermatophores short, 10.5 to 12.3 mm, around 1/3 ML and thin (2–3 mm, mean 2.75 mm); sperm reservoir around half of spermatophore length (50–55% ML), with sperm cord coiled in (48–56 mm, mean 54.5 mm); Cement and ejaculatory apparatus approximately 16% and 27% of spermatophore length respectively (Fig. 5c).

#### Female reproductive system

Maturing females with ovary occupying posterior half of mantle cavity, short proximal oviducts, oviducal glands with distinct radial chambers and longer distal oviducts (Fig. 5d); Eggs small, striated, almost mature ovarian eggs of ovaries 1.5–2.1 mm long, with median length 1.7 mm (Fig. 5d,e); Fecundity of 35 mm ML female estimated by gravimetric method at around 4000 eggs.

#### Etymology

The genus name is a combination of lepido from scale in Greek, referring to the scaly appearance given by the large almost overlapping papillae (dermal cushions) on its skin giving it a peculiar appearance as if it is covered with scales, and octopus. The name *joaquini* refers to the young son of the first author of this paper (JBL Sales).

## Discussion

The detailed morphological and genetic analyses presented here indicate that the red snapper, *Lutjanus purpureus*, can provide informative samples of the benthic and neritic benthopelagic cephalopods of the families Octopodidae and Loliginidae found in the southwestern Atlantic. In fact, the results of the present study indicate a much greater diversity of cephalopods in this region, in comparison with previous analyses. Barroso^41^ identified the genera *Octopus* and *Loligo* Lamarck, 1798 (now *Doryteuthis*) in the diet of *L. purpureus* (under the synonym *Lutjanus aya* Bloch, 1795) in specimens collected off northeastern Brazil. In the same region, Furtado-Ogawa & Menezes^30^ recorded a diet in this species (*L. purpureus*) dominated by fish, supplemented primarily by crustaceans and mollusks, including both loliginids and octopodids. In the diet of *Lutjanus analis* Cuvier, 1828 from Colombia, the only cephalopods identified by Duarte & Garcia^33^ were loliginid squid and octopuses of the genus *Octopus*. Fonseca^42^ found that the cephalopod prey of *Lutjanus synagris* Linnaeus, 1758 and *Ocyurus chrysurus* Bloch, 1791 collected in the Abrolhos Bank in the Brazilian state of Bahia included both octopodids and loliginids.

### Squids

The fishermen who provided the snapper specimens reported that squids were rarely found in the stomachs of these fish, and this is confirmed by the relatively small number of squid samples collected in the present study. The identification of both *Doryteuthis plei* and *Doryteuthis pealeii* in the samples analyzed was expected, given the known occurrence of these species in the study region^9^, and their use of demersal habitats^43^.

The genus *Abralia* Gray, 1849 has 19 species, and the specimen collected in the present study is likely to belong to one of the five species known to occur in the Atlantic Ocean, and specifically, to one of the two species for which no 16S sequences are available. While the present specimen was associated with the three species (*Abralia veranyi* Ruppel, 1844, *Abralia trigonura*, and *A. andamanica*) for which 16S sequences are available, and could thus be assigned to this genus, the uncorrected p-distance values were well above the levels used to identify cephalopods species^26,36^. This indicates that the specimen represents one of the other Atlantic species, either *Abralia redfieldi* Voss, 1955 or *Abralia grimpei* Voss, 1959^44^, for which no 16S sequences are available. *Abralia* are mesopelagic, upper slope squid that undertake diel vertical migration, remaining in deep water during the day (700–800 m in *A. veranyi*), and coming to the surface (40–60 m in *A. veranyi*) at night^44,45^. It is probably preyed on only occasionally by neritic lutjanids, and is likely to be captured only very rarely by any fishing technique.

### Octopods

#### *Macrotritopus* sp

No sequences (COI or 16S) are available for *Macrotritopus* in GenBank. The closest taxon with which the specimens collected in the present study were associated was “Blandopus” White V, designated “White V Octopus”, *Octopus* sp.18 by Norman^37^, and subsequently as “Blandopus” by Hanlon *et al*.^46^. Both *Macrotritopus* cf. *defilippi* and “Blandopus” White V is long-armed species known to mimic other organisms in a manner similar to *Thaumoctopus mimicus* Norman & Hochberg, 2005. In particular, *Macrotritopus* cf. *defilippi* (probably now the species more recently described as *M. beatrixi*) has been observed mimicking flatfish when moving over sandy substrates in the Caribbean^40^. The complete phylogeny based on the concatenated data (Fig. 3) clearly groups together all the mimetic octopuses, *Thaumoctopus mimicus*^47^, *Wunderpus photogenicus* Hochberg, Norman & Finn^48^, “Blandopus” white V^46^, and *Abdopus aculeatos*^49^ with high support values in general, with slightly weaker support for the association *Abdopus aculeatus* at the base of the clade.

#### *Octopus vulgaris* species complex

These samples were identified genetically as *Octopus vulgaris* from the southwestern Atlantic, and most closely related to the *O. vulgaris* types I and II of Jereb *et al*.^50^. While type II has only been reported previously from the southwestern Atlantic, Fig. 3b indicates the existence of well-supported clades in *O. vulgaris* that include sequences of samples from Brazil (including one from the same region as the present study - see Sales *et al*.^36^), France, the mid-Atlantic Island of São Paulo, the southeastern Indian Ocean, Turkey and Venezuela. The genus *Octopus* has been the subject of taxonomic revisions^5,6^, but the cryptic species complex of the western Atlantic *Octopus vulgaris* has only recently begun to be unraveled^51^, showing that *Octopus vulgaris* is composed of multiple *O. vulgaris*-like species that are currently being incorrectly treated under a single species name. Our concatenated data indicate the presence of more than one lineage of the *Octopus vulgaris* species complex along the Brazilian Coast. Including more individuals (not from stomach samples, and from other regions of Brazil) increases support values of all clades (Europe, South Africa, and two lineages from Brazil) in phylogenetic analyses (unpublished data). More individuals from along the South American coast (including from stomach contents) will help shed new light in this puzzle.

#### *Scaeurgus unicirrhus*

Species of the genus *Scaeurgus* are deep-water, benthic octopuses, found on different substrates of the continental shelf and on slopes at depths of between 100 m and 500 m^52,53^. *Scaeurgus* species are found in all tropical and temperate seas. Until recently, the genus was divided into only two species, distributed in the Atlantic (*S. unicirrhus*) and the Pacific (*Scaeurgus patagiatus* Berry, 1913) oceans. However, Kubodera & Lu^54^ found evidence of a potential new species off the coast of Taiwan, and Norman *et al*.^53^ described three new species - *S. tuber* Norman, Hochberg & Boucher-Rodoni, 2005*, S. jumeau* Norman, Hochberg & Boucher-Rodoni, 2005 and *S. nesisi* Norman, Hochberg & Boucher-Rodoni, 2005 - from New Caledonia and sea-mounts in the nearby Coral Sea.

The samples analyzed in the present study produced contrasting topologies. While the individual 16S and COI datasets indicated the existence of two *Scaeurgus* clades (Fig. 2), the concatenated data pointed to a single group. Given the discrete levels of p-distance divergence found between clades (0.0–1.2% in the 16S and 1.0% in COI), the results of these analyses are consistent with the presence of a single species with population structuring.

#### *Amphioctopus*

All *Amphioctopus* samples suffered a high degree of degradation from digestive processes and could only be identified by molecular techniques. *Amphioctopus* was recently revalidated in reviews of the genus *Octopus* (*Octopus aegina* group), and includes species targeted by commercial fisheries, especially in Southeast Asia^5,48,55^. *Amphioctopus burryi* (Voss, 1950) is the only species documented from the southwestern Atlantic Ocean. A single individual, identified only as *Amphioctopus* sp. (AmspPA86), was described from this region by Sales *et al*.^36^. The samples identified in the present study (151 and 152) were identical to AmspPA86. Together with well-preserved material obtained recently from Mexico and the Brazilian state of Rio de Janeiro, all known specimens from the region can be identified as *A. burryi* (Figs 2 and 3). Further analyses will nevertheless be necessary to confirm the phylogenetic position of this taxon in relation to the genera *Amphioctopus*, *Hapalochlaena* and *Callistoctopus* considering the polyphyletic characteristics of *Amphioctopus*, as determined by Dai *et al*.^26^.

#### *Lepidoctopus joaquini* Haimovici and Sales, gen. et sp. nov

This clade appears to be part of a weakly supported polytomic clade (0.84 posterior probability) including the genera *Eledone*, *Muusoctopus*, *Scaeurgus*, *Callistoctopus* (*sensu* Strugnell *et al*.^56^) and the species *O. rubescens* and *O. tehuelchus*, (Fig. 3)*. Octopus tehuelchus* has been considered more closely related to *Callistoctopus* than other *Octopus* species^57^. Based on the form of the hectocotylus of the better-preserved specimens, the new taxon appears to be more similar morphologically to *Callistoctopus*, which is represented by a single species in the eastern Atlantic (*Callistoctopus macropus*). However, the uncorrected p-distances of 12.6% from *Callistoctopus luteus* and 11.9% from *Callistoctopus minor*, indicate that the specimens are only distantly related to *Callistoctopus*, and, when compared with divergence values between described cephalopod genera, supports the hypothesis that these samples represent a different genus. Additionally, none of the other 21 genera within the Octopodidade^35^ share the morphological characters of dense dermal cushions and a long ligula with a longitudinal groove, supporting the generic status of this new taxon.

### Sampling cephalopod diversity through the analysis of stomach contents

The cephalopod diversity of South and Central America is still relatively poorly known, and the findings of the present study have further highlighted the ongoing trend of species descriptions over the past 60 years^8,58–60^, and the increasing evidence of genetic divergence along the Atlantic coast of the Americas^34^.

While many previous studies have used molecular tools to identify species to understand the diet of a particular predator species or increase sampling efficiency of specific known prey taxa^61,62^ none have used molecular studies of stomach contents as an approach for surveying the general biodiversity of a taxonomic group in relatively inaccessible habitats. The selection of a suitable predator that feeds in or around the target environment is fundamental to the success of this approach. In the present study, cephalopod samples were obtained from substrates that are not normally targeted by fisheries using gear suitable for the capture of these organisms. The sampling of stomach contents also circumvents the problem of vertical migrations of the target cephalopods, given that the predators may “sample” their prey at any time of day. While, this aggregative effect may imply imprecision of the sampling location, the ability to adequately identify morphological characters and produce DNA sequence data implies a degree of sample freshness and at least a moderate precision of the location. Although the digestive degradation of the specimens does limit formal taxonomic descriptions in some cases, repeated sampling may well provide more intact specimens suitable for morphological description and identification.

The use of a metagenomic approach to screen stomach contents rapidly for a range of prey organisms (e.g. Berry *et al*.^63^) should increase considerably the occurrence data for the target species. This approach should be complemented with classic morphological analyses, when possible, and individual molecular diagnoses, to provide a systematic record of the principal items in the diet of the predator, in addition to reference material for the description of new taxa.

This study demonstrated the potential of a combined morphological and molecular approach for the identification of the cephalopod fauna of areas that are difficult to sample directly. While many samples had been degraded by the digestive process, it was possible to obtain sequences from 100% of squid samples and approximately 63% of the octopod samples collected (14/14 squid and 82/130 octopods). This reinforces the potential of molecular tools for the identification of the species in aquatic food chains, as well as the understanding of the diversity of poorly-studied regions and hard-to-sample environments, such as the continental shelf and reef systems found off the mouth of the Amazon River.

## Methods

### Ethics statement

All methods were carried out in accordance with relevant guidelines and regulations. This included full license for the collection of biological material from already deceased animals (Under Brazilian biodiversity collection authorization license: SISBIO Permanent License 5857–1). As all material sampled in this project obtained from commercial fishermen was already dead, there was no requirement for ethical approval of sampling protocols as it did not include live organisms.

#### Sampling and sample identification

**Figure 6:**
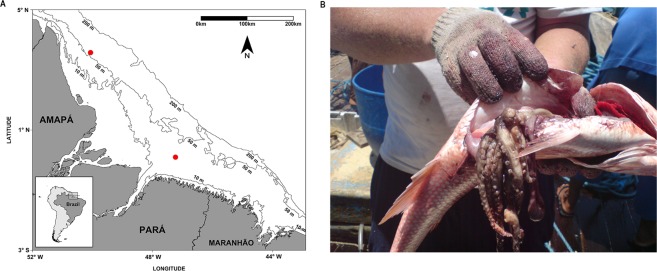
(**A**) Area in which the red snappers analyzed in the present study were captured. The red dots indicate the points at which the traps were set. (**B**) Cephalopods found in the stomachs of snappers recently captured within the study area. Amapá Coast sampling point: 00°05′53″N, 50°03′49″W. Pará State sampling point: 03°34′27″N, 47°14′08″W.

Partially digested and near-intact (which generally lacked the suckers or the tips of the tentacles) cephalopods were collected from the stomachs of recently fished red snapper, *Lutjanus purpureus*, between 2006 and 2008 at two fishing grounds in the continental shelf off the Amazon coast of the Brazilian states of Amapá and Pará (Fig. 6A). One area is located in the northern sector of the Amazon Reef System (00°05′53″N, 50°03′49″W) where the plume of the Amazon River flows primarily to the northwest, and the Photosynthetically Active Radiation (PAR) almost never reaches the bottom, even though the euphotic layer increases with increasing depth. The second area is located in the central sector of the reef system (03°34′27″N, 47°14′08″W), where the PAR often reaches the bottom, but with substantial spatial and temporal variation related to the dynamics of the Amazon plume, resulting in a shallow, mesophotic environment^28^.

The red snappers were caught by a small-scale artisanal fishery fleet based in the town of Bragança, Pará. Crew members confirmed fishing in areas at depths of up to 80 m over rocky and muddy substrates where nets cannot be used, and traps are frequently lost due to both to the characteristics of the substrate and the extremely turbulent currents. The cephalopod samples were washed immediately after collection to remove external contaminants, and then rinsed with diluted bleach to degrade any remaining red snapper DNA, washed again with water and then preserved directly in absolute ethanol for transportation to the laboratory in the town of Bragança (Fig. 6B). In total, 130 octopod samples and 14 squid samples were obtained during the study period. A small tissue sample was removed for molecular analyses, and the remaining specimen was fixed in a 10% formalin solution before being shipped to the Laboratory for Demersal Resources and Cephalopods at the Federal University of Rio Grande, Rio Grande do Sul state, Brazil for examination and identification based on external morphology. The complete list of specimens sampled are found in Supplementary Data 7.

As many of the squid specimens lost their skin, suckers, and part of their arms, they could only be identified morphologically to family (Loliginidae Lesueur, 1821). Some octopuses were better preserved, and it was possible to measure the length and width of the mantle, total length, the length and width of some arms, and the characteristics of the hectocotylized arm in males. In some cases, the diameter and the number of oocytes, the number and length of the spermatophores and the number of gill lamellae could also be determined, and a more detailed morphological description was possible.

#### Extraction, amplification and sequencing of the DNA

The DNA was extracted using three different protocols. The phenol-chloroform protocol^64^ was applied to samples that were relatively well-preserved. The DNA of the more degraded samples was extracted using either the Qiagen DNeasy kit (Valencia, CA) or the Invitrogen Pure Link Genomic DNA Mini kit (Carlsbad, CA), using the mouse tail protocol in both cases. The fragments of the mitochondrial 16S rRNA and Cytochrome Oxidase I (COI) genes were amplified by PCR using the procedure described by Sales *et al*.^36^ and the primers L1987/H2609 proposed by Palumbi *et al*.^65^ and LCO1490/HC02198 Folmer *et al*.^66^.

#### Molecular analyses

Sequences were aligned automatically using ClustalW^67^, run in BioEdit v.7.0.3^68^, and then visually inspected for errors. In cases of doubt, bases were called conservatively. Sequences were deposited in GenBank under the following accession numbers: For 16S squid**:** MG010606 - MG010619, for 16S octopus: MG010487–MG010544 and for COI Octopus: MG010545–MG010605. Only the 16S rRNA sequence (495 bps) was sequenced successfully in the case of squid samples, providing a single data set. For octopuses, both markers were amplified in only a subset of specimens, creating three distinct data sets: (i) 16S rRNA (541 bps), (ii) COI (659 bps), and (iii) the concatenated 16S and COI sequences. For the individual markers, GenBank sequences were only included in the analyses when similarity was at least 95%. For the concatenated data set, additional sequences representing all the genera of the family Octopodidae d’Orbigny, 1840 were obtained from GenBank to determine the most likely phylogenetic position of the samples that could not be identified morphologically. As all the BOLD sequences were also available in GenBank, only the GenBank sequences were included in the analyses. For all analyses, *Argonauta nodosa* Lightfoot, 1786 was used as outgroup.

MEGA 6.0^69^ was used to calculate uncorrected p-distances for all datasets to allow direct comparison across datasets and with published data. For production of phylogenetic trees, distances were based on the best evolutionary model selected for jModelTest2^70^ for each database. Neighbor-Joining (NJ) trees^71^ were produced, with support for the nodes based on 1000 bootstrap generations^72^. Maximum Likelihood (ML) and Bayesian Inference (BI) approaches were used to verify the clusters produced by the NJ trees and to analyze the concatenated dataset including all the species. The COI data set was partitioned (codon positions: 1 *vs*. 2 *vs*. 3; 1 + 2 *vs*. 3; and 1 + 2 + 3) based on Akaike Information Criterion (AIC), and ML trees produced using PhyML 3.0^73^ with support for nodes determined by 1000 bootstrap generations^74^. BI trees were produced in Mr. Bayes 3.2^75^, based on MCMC (Markov Chain Monte Carlo) sampling, with four simultaneous runs of 10 million generations, each consisting of four chains (one cold and three hot). Bayesian posterior probabilities were defined using a 70% consensus rule, random seeds, and sampling every 100 generations. To guarantee reliability, the first 25% of trees sampled in each MCMC run were discarded as burn-in. Log-likelihood scores were then plotted in Tracer v. 1.4^76^ to confirm the validity of the burn-in criterion. The post burn-in samples were used to construct a strict consensus tree.

#### Morphological analysis

All cephalopods collected from stomach contents were partly digested and lost parts of the arms and suckers. However, some were sufficiently preserved to allow some measurements and counts. Octopuses had their total weight (TW), total length (TL) dorsal mantle length (DML, measured from the posterior end to the midpoint between the eyes), mantle width (MW), funnel length (FL) free funnel length (FFL), the length (AL) and width (AW) of some arms and in males the hectocotylus (Hc), ligula length (LL) and calamus length (CL) were measured. In some cases, the diameter and number of intraovaric oocytes, the number and length of spermatophores and the number of gill lamellae could be measured or counted. All measurements were carried out following Roper and Voss^77^ and indices were expressed as quotients relative to the mantle length except for the free funnel index (FFI = FFL/FL) and the calamus index (CL/LL). Males with a fully developed hectocotylus and/or fully developed spermatophores in their spermatophoric sac and females with developed oviducal glands and/or developing oocytes in their ovaries were considered as mature for description purposes.

## Supplementary information

Supporting information
