# Supplementary material for: Surveying cephalopod diversity of the Amazon reef system using samples from red snapper stomachs and description of a new genus and species of octopus
Source: Sci Rep. 2019 Apr 11;9:5956. doi: 10.1038/s41598-019-42464-8 (PMC6459862; doi:10.1038/s41598-019-42464-8)
Supplement: Supplementary file 1 — Supporting information [file 41598_2019_42464_MOESM1_ESM.docx]

**Supplementary information**

Surveying cephalopod diversity of the Amazon reef system using samples from red snapper stomachs and description of a new genus and species of octopus.

João Bráullio de Luna Sales^1,3*^, Manuel Haimovici^2^, Jonathan Stuart Ready^3^, Rosália Furtado Souza^4^, Yrlene Ferreira^5^, Jessica de Cassia Silva Pinon^6^, Luis Fernando Carvalho Costa^7^, Nils Edvin Asp^8^, Iracilda Sampaio^5^ and Horacio Schneider^†5^

**Supplementary data 1**: Uncorrected p-distancesfor 16S data between squid samples collected for the present study with comparison of GenBank sequences;

A-*Doryteuthis plei*

|  | 1 | 2 | 3 | 4 | 5 | 6 | 7 | 8 | 9 | 10 | 11 | 12 | 13 | 14 | 15 | 16 | 17 |
| --- | --- | --- | --- | --- | --- | --- | --- | --- | --- | --- | --- | --- | --- | --- | --- | --- | --- |
| 1.Dple30K854032 |  |  |  |  |  |  |  |  |  |  |  |  |  |  |  |  |  |
| 2.*Loligo plei* AF110080 | 0.010 |  |  |  |  |  |  |  |  |  |  |  |  |  |  |  |  |
| 3.Dple212 KF854025 | 0.007 | 0.002 |  |  |  |  |  |  |  |  |  |  |  |  |  |  |  |
| 4.Dple443 KF854012 | 0.048 | 0.043 | 0.041 |  |  |  |  |  |  |  |  |  |  |  |  |  |  |
| 5.Dple357 KF854013 | 0.048 | 0.043 | 0.041 | 0.000 |  |  |  |  |  |  |  |  |  |  |  |  |  |
| 6.Sample squid37 | 0.048 | 0.043 | 0.041 | 0.002 | 0.002 |  |  |  |  |  |  |  |  |  |  |  |  |
| 7.Sample squid31 | 0.048 | 0.043 | 0.041 | 0.002 | 0.002 | 0.000 |  |  |  |  |  |  |  |  |  |  |  |
| 8.Sample squid25 | 0.048 | 0.043 | 0.041 | 0.000 | 0.000 | 0.002 | 0.002 |  |  |  |  |  |  |  |  |  |  |
| 9.Sample squid32 | 0.051 | 0.046 | 0.043 | 0.002 | 0.002 | 0.005 | 0.005 | 0.002 |  |  |  |  |  |  |  |  |  |
| 10.Sample squid28 | 0.048 | 0.043 | 0.041 | 0.000 | 0.000 | 0.002 | 0.002 | 0.000 | 0.002 |  |  |  |  |  |  |  |  |
| 11.Sample squid56 | 0.048 | 0.043 | 0.041 | 0.000 | 0.000 | 0.002 | 0.002 | 0.000 | 0.002 | 0.000 |  |  |  |  |  |  |  |
| 12.Sample squid53 | 0.048 | 0.043 | 0.041 | 0.000 | 0.000 | 0.002 | 0.002 | 0.000 | 0.002 | 0.000 | 0.000 |  |  |  |  |  |  |
| 13.Sample squid30 | 0.048 | 0.043 | 0.041 | 0.000 | 0.000 | 0.002 | 0.002 | 0.000 | 0.002 | 0.000 | 0.000 | 0.000 |  |  |  |  |  |
| 14.Sample squid34 | 0.048 | 0.043 | 0.041 | 0.000 | 0.000 | 0.002 | 0.002 | 0.000 | 0.002 | 0.000 | 0.000 | 0.000 | 0.000 |  |  |  |  |
| 15.Sample squid24 | 0.048 | 0.043 | 0.041 | 0.000 | 0.000 | 0.002 | 0.002 | 0.000 | 0.002 | 0.000 | 0.000 | 0.000 | 0.000 | 0.000 |  |  |  |
| 16.Sample squid40 | 0.051 | 0.046 | 0.043 | 0.002 | 0.002 | 0.005 | 0.005 | 0.002 | 0.005 | 0.002 | 0.002 | 0.002 | 0.002 | 0.002 | 0.002 |  |  |
| 17.Sample squid26 | 0.048 | 0.043 | 0.041 | 0.000 | 0.000 | 0.002 | 0.002 | 0.000 | 0.002 | 0.000 | 0.000 | 0.000 | 0.000 | 0.000 | 0.000 | 0.002 | - |

B-*Doryteuthis pealeii*

|  | 1 | 2 | 3 | 4 | 5 | 6 | 7 |
| --- | --- | --- | --- | --- | --- | --- | --- |
| 1.Dpea118 KF854015 |  |  |  |  |  |  |  |
| 2.Dpea119 KF854016 | 0.000 |  |  |  |  |  |  |
| 3.Dpea131 KF854017 | 0.000 | 0.000 |  |  |  |  |  |
| 4.Sample squid58 | 0.000 | 0.000 | 0.000 |  |  |  |  |
| 5.*Loligo pealeii* AF110079 | 0.014 | 0.014 | 0.014 | 0.014 |  |  |  |
| 6.*Loligo pealeii* AY686590 | 0.014 | 0.014 | 0.014 | 0.014 | 0.005 |  |  |
| 7.Dpea12 KF854026 | 0.011 | 0.011 | 0.011 | 0.011 | 0.002 | 0.002 | - |

C-*Abralia*

|  | 1 | 2 | 3 | 4 |
| --- | --- | --- | --- | --- |
| 1.Sample squid19 |  |  |  |  |
| 2.*Abralia adamanica* HQ855987 | 0.046 |  |  |  |
| 3.*Abralia veranyi* EU735259 | 0.057 | 0.044 |  |  |
| 4.*Abralia trigonura* X79584 | 0.062 | 0.062 | 0.044 |  |

**Supplementary data 2**: Uncorrected p-distances between octopus samples collected for the present study in comparison with the closest matchingGenBank sequences found by BLAST searches;

A-*Macrotriptopus* clade -16S

|  | 1 | 2 | 3 | 4 | 5 |
| --- | --- | --- | --- | --- | --- |
| 1.Blandopus whiteV GQ900724 |  |  |  |  |  |
| 2.Sample octo52 | 0.044 |  |  |  |  |
| 3.Sample octo53 | 0.044 | 0.000 |  |  |  |
| 4.Sample octo55 | 0.047 | 0.003 | 0.003 |  |  |
| 5.Sample octo65* | 0.052 | 0.008 | 0.008 | 0.010 |  |

* A single haplotype representing identical sequences from samples:54, 64, 65, 74,82, 83, 96, 98, 99;

B-*Macrotritopus* clade - COI

|  | 1 | 2 | 3 | 4 | 5 | 6 | 7 | 8 | 9 |
| --- | --- | --- | --- | --- | --- | --- | --- | --- | --- |
| 1.Blandopus white V GQ900747 |  |  |  |  |  |  |  |  |  |
| 2.Sample octo85 | 0.055 |  |  |  |  |  |  |  |  |
| 3. Sample octo226 | 0.051 | 0.007 |  |  |  |  |  |  |  |
| 4 Sample octo97 | 0.051 | 0.004 | 0.002 |  |  |  |  |  |  |
| 5.Sample octo101 | 0.051 | 0.009 | 0.007 | 0.004 |  |  |  |  |  |
| 6.Sample octo106 | 0.051 | 0.007 | 0.004 | 0.002 | 0.007 |  |  |  |  |
| 7.Sample octo108 | 0.049 | 0.009 | 0.002 | 0.004 | 0.009 | 0.007 |  |  |  |
| 8.Sample octo53 | 0.053 | 0.007 | 0.004 | 0.002 | 0.002 | 0.004 | 0.007 |  |  |
| 9.Sample octo82 | 0.053 | 0.007 | 0.004 | 0.002 | 0.002 | 0.004 | 0.007 | 0.000 |  |
| 10.Sample octo55* | 0.049 | 0.007 | 0.004 | 0.002 | 0.007 | 0.002 | 0.007 | 0.004 | 0.004 |

*A single haplotype representing identical sequences from samples: 52, 54, 64, 65, 66, 67, 68, 71, 74, 75, 83, 91, 92, 96, 98, 99, 100, 102, 103,105;

C-*Octopus vulgaris* species complex clade *-* 16S

|  | 1 | 2 | 3 | 4 | 5 | 6 |
| --- | --- | --- | --- | --- | --- | --- |
| 1.*O. vulgaris* AJ252770 |  |  |  |  |  |  |
| 2.*O. vulgaris* AJ390316 | 0.005 |  |  |  |  |  |
| 3.OvuPA 78 KF843974 | 0.009 | 0.009 |  |  |  |  |
| 4.OvuPA79 KF843973 | 0.009 | 0.009 | 0.000 |  |  |  |
| 5.OvuPA173 KF843972 | 0.011 | 0.007 | 0.016 | 0.016 |  |  |
| 6.OvuAP225 KF843970* | 0.002 | 0.002 | 0.007 | 0.007 | 0.009 |  |
| 7.Sample octo161 | 0.009 | 0.005 | 0.014 | 0.014 | 0.002 | 0.007 |

*A single haplotype representing identical sequences from samples: 62, 63, 69, 70, 81, 84, 87, 88, 89, 90, 94, 95, 104, 107, 109, 111, 155, 156, 164,184;

D-*Octopus vulgaris* species complex clade *-* COI

|  | 1 | 2 | 3 | 4 | 5 | 6 | 7 | 8 |
| --- | --- | --- | --- | --- | --- | --- | --- | --- |
| 1.*O. vulgaris*_KJ605279 |  |  |  |  |  |  |  |  |
| 2.*O. vulgaris_*KC311412 | 0.000 |  |  |  |  |  |  |  |
| 3.*O. vulgaris*_EF016328 | 0.000 | 0.000 |  |  |  |  |  |  |
| 4.*O.vulgaris*_FN424379 | 0.000 | 0.000 | 0.000 |  |  |  |  |  |
| 5. *O. vulgaris*_FN424380 | 0.000 | 0.000 | 0.000 | 0.000 |  |  |  |  |
| 6.OvuPA173_KF8404027 | 0.000 | 0.000 | 0.000 | 0.000 | 0.000 |  |  |  |
| 7.OvuPA184_KF844030 | 0.016 | 0.016 | 0.016 | 0.016 | 0.016 | 0.016 |  |  |
| 8. OvuRJ_131_KF844033 | 0.021 | 0.021 | 0.021 | 0.021 | 0.021 | 0.021 | 0.005 |  |
| 9. Sample_octo225* | 0.021 | 0.021 | 0.021 | 0.021 | 0.021 | 0.021 | 0.005 | 0.000 |

*A single haplotype representing identical sequences from samples:63, 69, 70, 78, 79;

E-*Scaeurgus* clade - 16S

|  | 1 | 2 | 3 | 4 | 5 |
| --- | --- | --- | --- | --- | --- |
| 1. *Scaeurgus_unicirrhus*_HM104248 | 0.000 |  |  |  |  |
| 2. *Scaeurgus_unicirrhus*_AJ390324 | 0.000 | 0.000 |  |  |  |
| 3. Sample_octo56 | 0.000 | 0.000 | 0.000 |  |  |
| 4. Sample_octo57 | 0.012 | 0.012 | 0.012 | 0.012 |  |
| 5. Sample_octo58* | 0.000 | 0.000 | 0.000 | 0.012 | 0.000 |

*A single haplotype representing identical sequences from samples 58F, 59, 60, 61

F-*Scaeurgus* clade - COI

|  | 1 | 2 |
| --- | --- | --- |
| 1.*Scaeurgus_unicirrhus*_HM104263 |  |  |
| 2. Sample_octo56 | 0.010 |  |
| 3. Sample_octo57* | 0.010 | 0.000 |

*A single haplotype representing identical sequences from samples 58, 61

G-*Amphioctopuis* clade *-* 16S

|  | 1 | 2 | 3 |
| --- | --- | --- | --- |
| 1. *Amphioctopus_aegina*_AB191111 |  |  |  |
| 2. *Amphioctopus_marginatus*_GQ900709 | 0.027 |  |  |
| 3. AmspPA86_KF843997 | 0.021 | 0.019 |  |
| 4. Sample_octo151* | 0.008 | 0.024 | 0.005 |

*A single haplotype representing identical sequence for sample 152

H-*Amphioctopuis* clade *-* COI

|  | 1 | 2 | 3 | 4 |
| --- | --- | --- | --- | --- |
| 1.*Amphioctopus_aegina*_JX456265 |  |  |  |  |
| 2.*Amphioctopus_marginatus*_HQ846139 | 0.005 |  |  |  |
| 3.AmspPA86_KF844045 | 0.042 | 0.042 |  |  |
| 4.Sample_octo72 | 0.040 | 0.040 | 0.005 |  |
| 5.Sample_octo185* | 0.042 | 0.042 | 0.002 | 0.002 |

*A single haplotype representing identical sequences from samples:151, 152

I-*Lepidoctopus*clade- 16S

|  | 1 | 2 | 3 | 4 | 5 | 6 | 7 | 8 |
| --- | --- | --- | --- | --- | --- | --- | --- | --- |
| 1.*Octopus_rubescens*_AJ252755 |  |  |  |  |  |  |  |  |
| 2. Sample_octo154 | 0.120 |  |  |  |  |  |  |  |
| 3. Sample_octo157 | 0.120 | 0.000 |  |  |  |  |  |  |
| 4. Sample_octo159 | 0.120 | 0.000 | 0.000 |  |  |  |  |  |
| 5. Sample_octo160 | 0.120 | 0.000 | 0.000 | 0.000 |  |  |  |  |
| 6. Sample_octo175 | 0.122 | 0.002 | 0.002 | 0.002 | 0.002 |  |  |  |
| 7. Sample_octo177 | 0.122 | 0.002 | 0.002 | 0.002 | 0.002 | 0.000 |  |  |
| 8. Sample_octo180 | 0.120 | 0.000 | 0.000 | 0.000 | 0.000 | 0.002 | 0.002 |  |
| 9. Sample_octo110 | 0.120 | 0.000 | 0.000 | 0.000 | 0.000 | 0.002 | 0.002 | 0.000 |

**Supplementary data 3:** A preserved 30 mm mantle length female of *Lepidoctopusjoaquini*gen. et sp. nov. (paratype MORG 51456) collected from the stomach of a snapper captured in coastal waters of northern Brazil (00°05’53” N, 50°03’49” W).


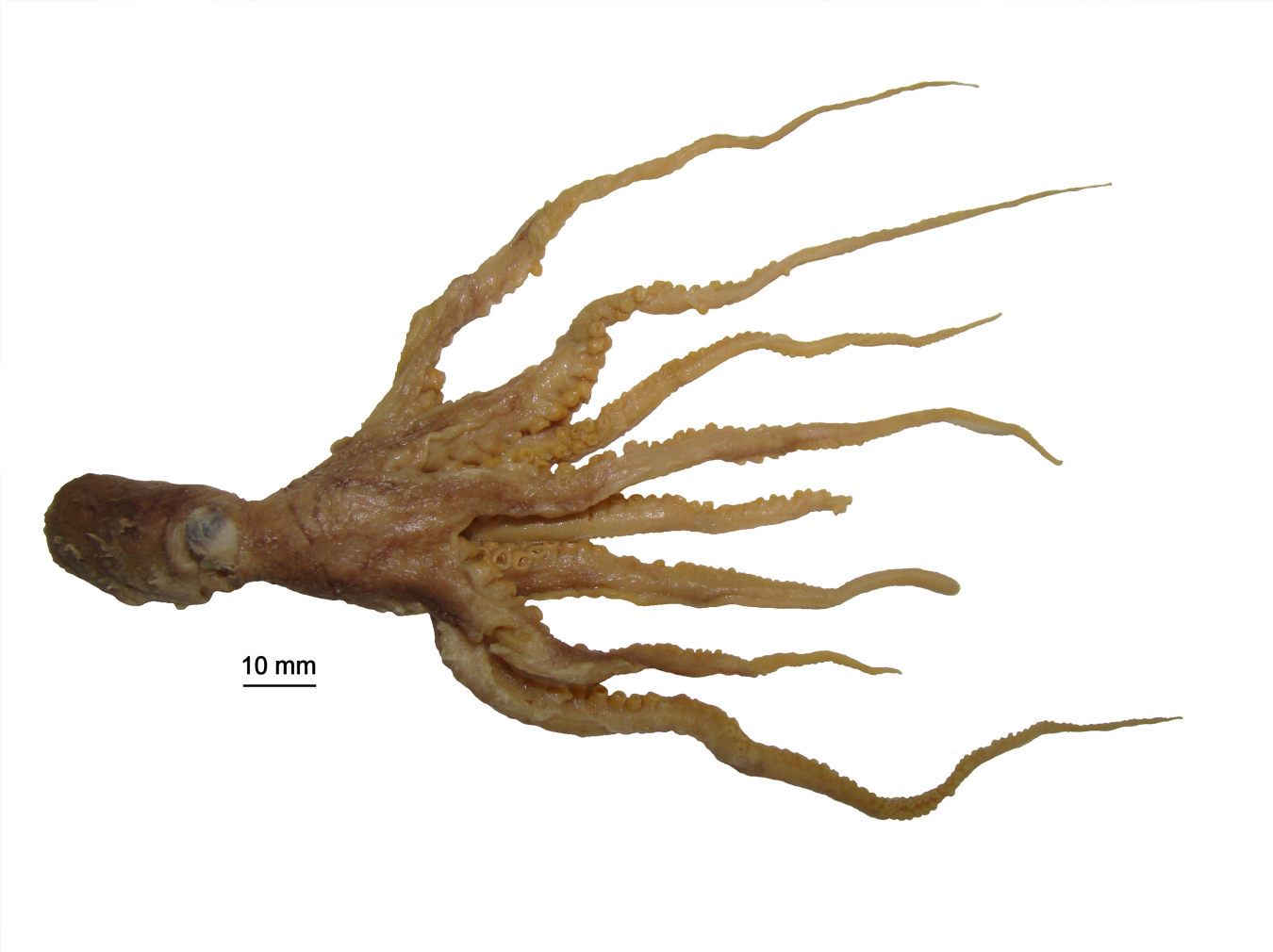


**Supplementary data 4:** A preserved 28 mm mantle length male of *Lepidoctopusjoaquini*gen. et sp. nov. (holotype MORG 51455) collected from the stomach of a snapper captured in coastal waters of northern Brazil (00°05’53” N, 50°03’49” W).


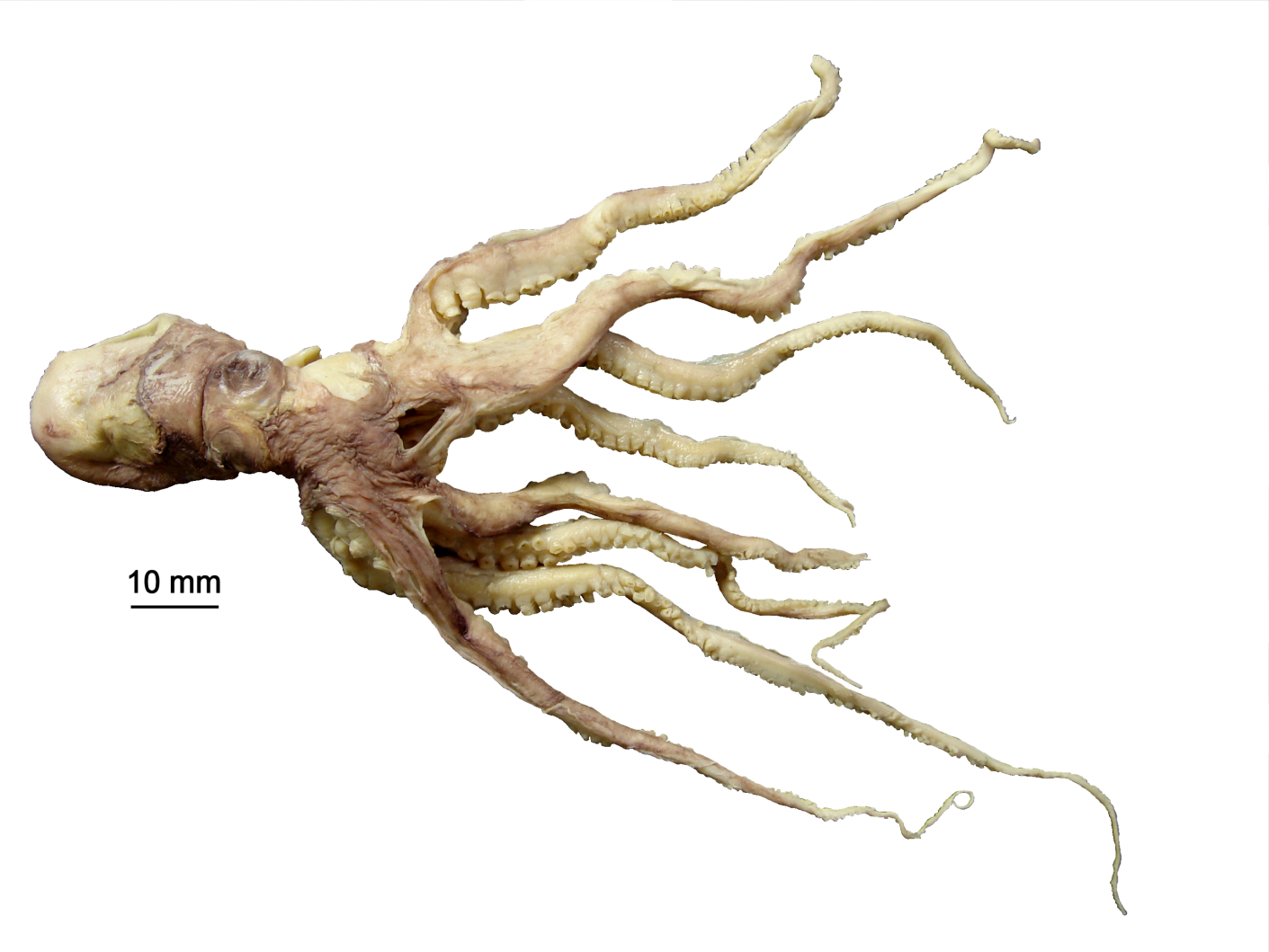


**Supplementary data 5:** Detail of the dermal papillae on the mantle of a preserved 30 mm mantle length female of *Lepidoctopusjoaquini*gen. et sp. nov. (paratype MORG 51456) collected from the stomach of a snapper captured in coastal waters of northern Brazil (00°05’53” N, 50°03’49” W).


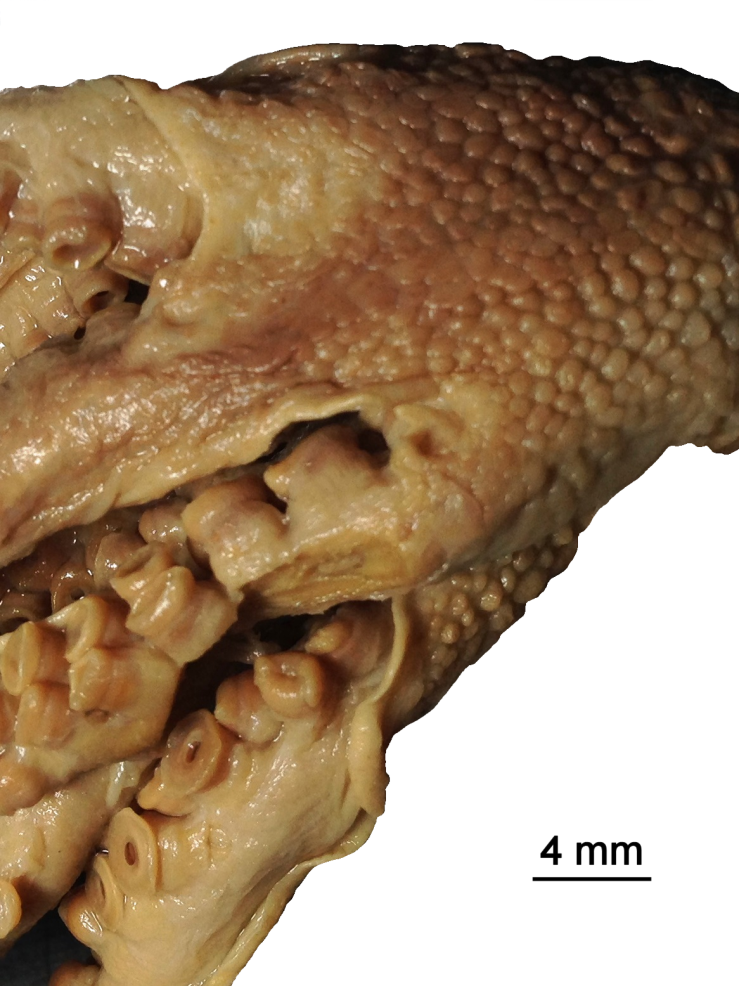


**Supplementary data 6:** Detail of the hectocotylus of a 28 mm mantle length male of *Lepidoctopusjoaquini*gen. et sp. nov.(holotype MORG 51455) collected from the stomach of a snapper captured in coastal waters of northern Brazil (00°05’53” N, 50°03’49” W.

**
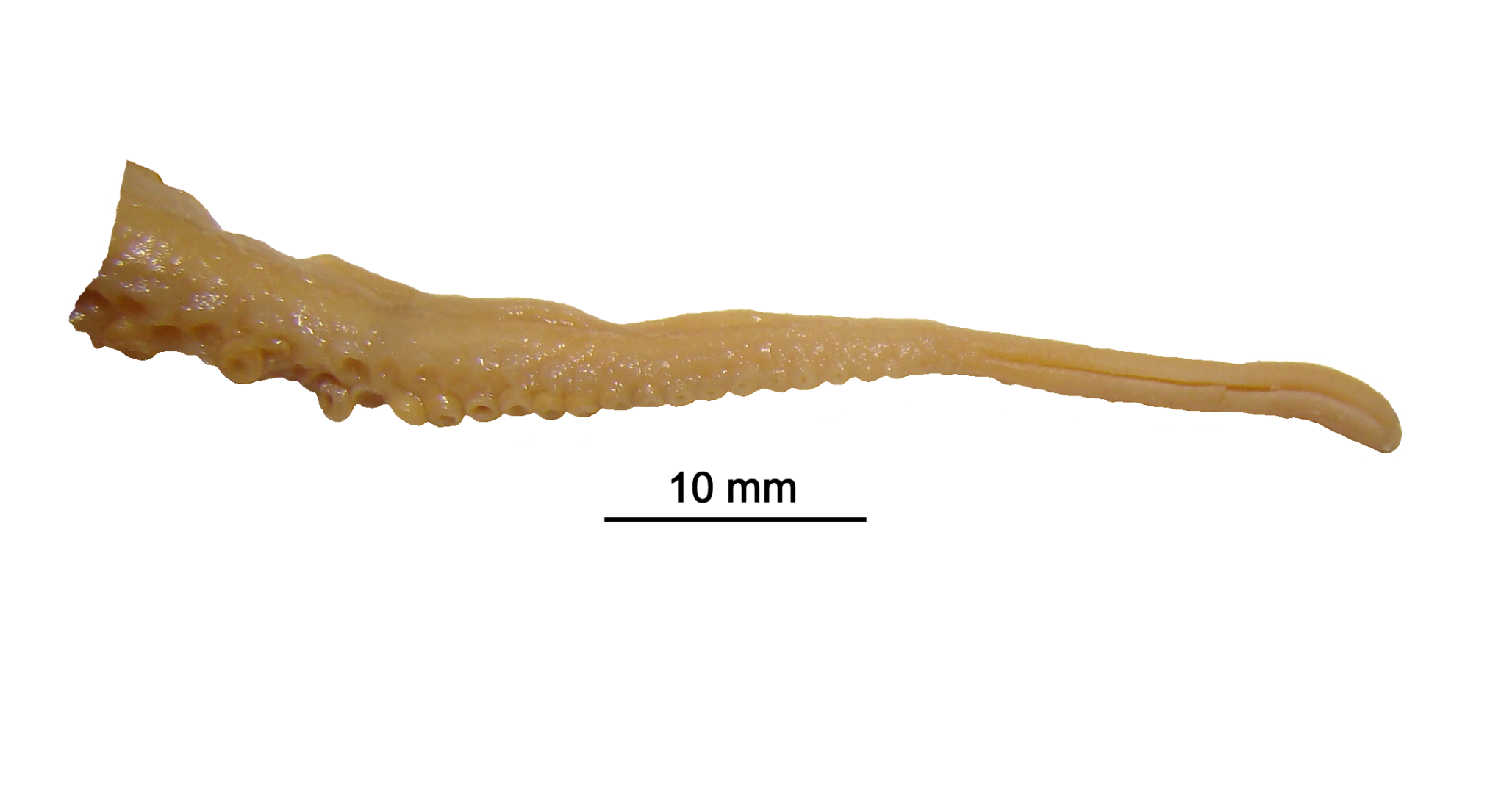
**

**Supporting data 7**: List of the samples collected with their respective codes, collecting site, and the percentage similarity of the sequences recorded in the present study with those retrieved online for the COI (GenBank and BOLD) and 16S (GenBank) genes.

| **SampleIdentification Code** | **GenBank accession number (16S/COI)** | **Sampling Place** | **Molecular Similarity: COI (GeneBank/BOLD)** | **Molecular Similarity: 16S (GeneBank)** |
| --- | --- | --- | --- | --- |
| Sample octo52 | MG010487/MG010554 | Amapá Coast | 96% similar to *Blandopus white* V/ No match | 95% similar to *Blandopus white V* |
| Sample octo53 | MG010488/MG010551 | Amapá Coast | 96% similar to *Blandopus white* V/ No match | 95% similar to *Blandopus white V* |
| Sample octo54 | MG010489/MG010555 | Amapá Coast | 96% similar to *Blandopus white* V/ No match | 95% similar to *Blandopus white V* |
| Sample octo55 | MG010490/MG010553 | Amapá Coast | 96% similar to *Blandopus white* V/ No match | 95% similar to *Blandopus white V* |
| Sample octo56V | MG010525/MG010581 | Amapá Coast | 99% Similar to *Scaeurgus unicirrhus*/ 98.6% similar to *Scaeurgus unicirrhus* | 99% similar to *Scaeurgus unicirrhus* |
| Sample octo57 | MG010526/MG010582 | Amapá Coast | 99% Similar to *Scaeurgus unicirrhus*/ 98.6% similar to *Scaeurgus unicirrhus* | 99% similar to *Scaeurgus unicirrhus* |
| Sample octo58 | MG010527/MG010583 | Pará Coast | 99% Similar to *Scaeurgus unicirrhus*/ 98.6% similar to *Scaeurgus unicirrhus* | 99% similar to *Scaeurgus unicirrhus* |
| Sample octo58 F | MG010528/MG010585 | Amapá Coast | 99% Similar to *Scaeurgus unicirrhus*/ 98.6% similar to *Scaeurgus unicirrhus* | 99% similar to *Scaeurgus unicirrhus* |
| Sample octo59 | MG010529/MG010586 | Pará Coast | 99% Similar to *Scaeurgus unicirrhus*/ 98.6% similar to *Scaeurgus unicirrhus* | 99% similar to *Scaeurgus unicirrhus* |
| Sample octo60 | MG010530/MG010587 | Pará Coast | 99% Similar to *Scaeurgus unicirrhus*/ 98.6% similar to *Scaeurgus unicirrhus* | 99% similar to *Scaeurgus unicirrhus* |
| Sample octo61 | MG010531/MG010584 | Amapá Coast | 99% Similar to *Scaeurgus unicirrhus*/ 98.6% similar to *Scaeurgus unicirrhus* | 99% similar to *Scaeurgus unicirrhus* |
| Sample octo62 | MG010503/- | Amapá Coast | - | 100% similar to *Octopus vulgaris* |
| Sample octo63 | MG010504/MG010578 | Amapá Coast | 100% similar to *Octopus vulgaris* / 97.4% similar to *Octopus vulgaris* | 100% similar to *Octopus vulgaris* |
| Sample octo64 | MG010491/MG010556 | Amapá Coast | 96% similar to *Blandopus white V* / No match | 95% similar to *Blandopus white V* |
| Sample octo65 | MG010492/MG010570 | Amapá Coast | 96% similar to *Blandopus white V* / No match | 95% similar to *Blandopus white V* |
| Sample octo66 | MG010557 | Amapá Coast | 96% similar to *Blandopus white V* / No match | - |
| Sample octo67 | MG010571 | Amapá Coast | 96% similar to *Blandopus white V* / No match | - |
| Sample octo68 | MG010572 | Amapá Coast | 96% similar to *Blandopus white V* / No match | - |
| Sample octo69 | MG010505/MG010577 | Amapá Coast | 99% similar to *Octopus vulgaris /* 97.4% similar to *Octopus vulgaris* | 99% similar to *Octopus vulgaris* |
| Sample octo70 | MG010506/MG010579 | Amapá Coast | 99% similar to *Octopus vulgaris /* 97.4% similar to *Octopus vulgaris* | 99% similar to *Octopus vulgaris* |
| Sample octo71 | MG010573 | Amapá Coast | 96% similar to *Blandopus white V* / No match | - |
| Sample octo72 | MG010588 | Amapá Coast | 95% similar to *Amphioctopus marginatus*/ No match | - |
| Sample octo74 | MG010493/MG010558 | Amapá Coast | 96% similar to *Blandopus white V* / No match | 95% similar to *Blandopus white V* |
| Sample octo75 | MG010534/MG010564 | Amapá Coast | 96% similar to *Blandopus white V* / No match | 92% similar to *Thaumoctopus mimicus*; |
| Sample octo77 | MG010524/MG010580 | Amapá Coast | 99% similar to *Octopus hummlincki/* No match | 99% similar to *Octopus hummlincki* |
| Sample octo78 | KF843973/MG010575 | Pará Coast | 99% similar to *Octopus vulgaris /* 97.7% similar to *Octopus vulgaris* | 99% similar to *Octopus vulgaris** |
| Sample octo79 | MG010544/MG010576 | Pará Coast | 99% similar to *Octopus vulgaris /* 97.7% similar to *Octopus vulgaris* | 99% similar to *Octopus vulgaris** |
| Sample octo81 | MG010507 | Amapá Coast | - | 100% similar to *Octopus vulgaris* |
| Sample octo82 | MG010494/MG010552 | Amapá Coast | 96% similar to *Blandopus white V* / No match | 95% similar to *Blandopus white V* |
| Sample octo83 | MG010495/MG010559 | Amapá Coast | 96% similar to *Blandopus white V* / No match | 92% similar to *Thaumoctopus mimicus*; |
| Sample octo84 | MG010508 | Amapá Coast | - | 99% similar to *Octopus vulgaris* |
| Sample octo85 | MG010545 | Amapá Coast | 96% similar to *Blandopus white V* / No match | - |
| Sample octo87 | MG010509 | Amapá Coast | - | 99% similar to *Octopus vulgaris* |
| Sample octo88 | MG010510 | Amapá Coast | - | 99% similar to *Octopus vulgaris* |
| Sample octo89 | MG010511 | Amapá Coast | - | 99% similar to *Octopus vulgaris* |
| Sample octo90 | MG010512 | Amapá Coast | - | 99% similar to *Octopus vulgaris* |
| Sample octo91 | MG010535/MG010563 | Amapá Coast | 96% similar to *Blandopus white V* / No match | 95% similar to *Blandopus white V* |
| Sample octo92 | MG010536/MG010565 | Amapá Coast | 96% similar to *Blandopus white V* / No match | 92% similar to *Thaumoctopus mimicus*; |
| Sample octo94 | MG010513 | Amapá Coast | - | 99% similar to *Octopus vulgaris* |
| Sample octo95 | MG010514 | Amapá Coast | - | 99% similar to *Octopus vulgaris* |
| Sample octo96 | MG010496/MG010560 | Amapá Coast | 96% similar to *Blandopus white V* / No match | 92% similar to *Thaumoctopus mimicus* |
| Sample octo97 | MG010547 | Amapá Coast | 96% similar to *Blandopus white V* / No match | - |
| Sample octo98 | MG010497/MG010568 | Amapá Coast | 96% similar to *Blandopus white V* / No match | 92% similar to *Thaumoctopus mimicus*; |
| Sample octo99 | MG010498/MG010566 | Amapá Coast | 96% similar to *Blandopus white V* / No match | 92% similar to *Thaumoctopus mimicus*; |
| Sample octo100 | MG010537/MG010561 | Amapá Coast | 96% similar to *Blandopus white V* / No match | 92% similar to *Thaumoctopus mimicus*; |
| Sample octo101 | MG010538/MG010548 | Amapá Coast | 96% similar to *Blandopus white V* / No match | 92% similar to *Thaumoctopus mimicus*; |
| Sample octo102 | MG010539/MG010569 | Amapá Coast | 96% similar to *Blandopus white V* / No match | 92% similar to *Thaumoctopus mimicus*; |
| Sample octo103 | MG010540/MG010567 | Amapá Coast | 96% similar to *Blandopus white V* / No match | 92% similar to *Thaumoctopus mimicus*; |
| Sample octo104 | MG010515 | Amapá Coast | - | 99% similar to *Octopus vulgaris* |
| Sample octo105 | MG010562 | Amapá Coast | 96% similar to *Blandopus white V* / No match | - |
| Sample octo106 | MG010549 | Amapá Coast | 96% similar to *Blandopus white V* / No match | - |
| Sample octo107 | MG010516 | Amapá Coast | - | 99% similar to *Octopus vulgaris* |
| Sample octo108 | MG010550 | Amapá Coast | 96% similar to *Blandopus white V* / No match | - |
| Sample octo109 | MG010517 | Amapá Coast | - | 99% similar to *Octopus vulgaris* |
| Sample octo110 | MG010541 | Amapá Coast | - | 91% similar to *Octopus rubescens* |
| Sample octo111 | MG010518 | Amapá Coast | - | 99% similar to *Octopus vulgaris* |
| Sample octo112 | MG010601 | Amapá Coast | 87% similar to *Octopus tehuelcus*/ No match | - |
| Sample octo137 | MG010602 | Pará Coast | 87% similar to *Octopus tehuelcus*/ No match | - |
| Sample octo139 | MG010603 | Pará Coast | 87% similar to *Octopus tehuelcus*/ No match | - |
| Sample octo140 | MG010604 | Pará Coast | 87% similar to *Octopus tehuelcus*/ No match | - |
| Sample octo151 | MG010532/MG010590 | Amapá Coast | 96% similar to *Amphioctopus marginatus/* No match | 98% simiar to *Octopus kogashimensis and Amphioctopus marginatus* |
| Sample octo152 | MG010533/MG010591 | Amapá Coast | 96% similar to *Amphioctopus marginatus/* No match | 98% simiar to *Octopus kogashimensis and Amphioctopus marginatus* |
| Sample octo154 | MG010520 | Amapá Coast | - | 91% similar to *Octopus rubescens* |
| Sample octo155 | MG010499 | Amapá Coast | - | 99% similar to *Octopus vulgaris* |
| Sample octo156 | MG010500 | Amapá Coast | - | 100% similar to *Octopus vulgaris* |
| Sample octo157 | MG010521 | Amapá Coast | - | 91% similar to *Octopus rubescens* |
| Sample octo159 | MG010522 | Amapá Coast | - | 91% similar to *Octopus rubescens* |
| Sample octo160 | MG010523 | Amapá Coast | - | 91% similar to *Octopus rubescens* |
| Sample octo161 | MG010501 | Amapá Coast | - | 99% similar to *Octopus vulgaris* |
| Sample octo164 | MG010502 | Amapá Coast | - | 100% similar to *Octopus vulgaris* |
| Sample octo173 | KF843972/KF844027 | Pará Coast | 99% similar to *Octopus vulgaris**/99.75% similar to *Octopus vulgaris* | 99% similar to *Octopus vulgaris* |
| Sample octo175 | MG010542/MG010592 | Pará Coast | 87% similar to *Octopus tehuelcus**/No match | 91% similar to *Octopus rubescens* |
| Sample octo177 | MG010543/MG010592 | Pará Coast | 87% similar to *Octopus tehuelcus**/No match | 91% similar to *Octopus rubescens* |
| Sample octo180 | MG010544/MG010593 | Pará Coast | 87% similar to *Octopus tehuelcus**/No match | 91% similar to *Octopus rubescens* |
| Sample octo182 | MG010594 | Pará Coast | 87% similar to *Octopus tehuelcus**/No match | - |
| Sample octo184 | MG010519/KF844030 | Pará Coast | 100% similar to *Octopus vulgaris**/98.23% similar to *Octopus vulgaris* | 99% similar to *Octopus vulgaris* |
| Sample octo185 | MG010589 | Pará Coast | 95% similar to *Amphioctopus marginatus/* No match | - |
| Sample octo186 | MG010595 | Pará Coast | 87% similar to *Octopus tehuelcus**/No match | - |
| Sample octo188 | MG010596 | Pará Coast | 87% similar to *Octopus tehuelcus**/No match | - |
| Sample octo190 | MG010597 | Pará Coast | 87% similar to *Octopus tehuelcus**/No match | - |
| Sample octo191 | MG010598 | Pará Coast | 87% similar to *Octopus tehuelcus**/No match | - |
| Sample octo192 | MG010599 | Pará Coast | 87% similar to *Octopus tehuelcus**/No match | - |
| Sample octo226 | MG010546 | Amapá Coast | 96% similar to *Blandopus white V* /No match | - |
| Sample squid19 | MG010606 | Amapá Coast | - | 95% similar to *Abralia* sp |
| Sample squid24 | MG010607 | Amapá Coast | - | 100% similar to *Doryteuthis plei* |
| Sample squid25 | MG010608 | Amapá Coast | - | 100% similar to *Doryteuthis plei* |
| Sample squid26 | MG010609 | Amapá Coast | - | 100% similar to *Doryteuthis plei* |
| Sample squid28 | MG010610 | Amapá Coast | - | 100% similar to *Doryteuthis plei* |
| Sample squid30 | MG010611 | Amapá Coast | - | 100% similar to *Doryteuthis plei* |
| Sample squid31 | MG010612 | Amapá Coast | - | 100% similar to *Doryteuthis plei* |
| Sample squid32 | MG010613 | Amapá Coast | - | 100% similar to *Doryteuthis plei* |
| Sample squid34 | MG010614 | Amapá Coast | - | 100% similar to *Doryteuthis plei* |
| Sample squid37 | MG010615 | Amapá Coast | - | 100% similar to *Doryteuthis plei* |
| Sample squid40 | MG010616 | Amapá Coast | - | 100% similar to *Doryteuthis plei* |
| Sample squid53 | MG010617 | Amapá Coast | - | 100% similar to *Doryteuthis plei* |
| Sample squid56 | MG010618 | Amapá Coast | - | 100% similar to *Doryteuthis plei* |
| Sample squid58 | MG010619 | Amapá Coast | - | 99% similar to *Doryteuthis pealei* |
